# Synergy of Oxygen and Water in Ceria-Catalyzed Direct Conversion of Methane to Methanol under Continuous Flow

**DOI:** 10.1021/acscatal.5c05829

**Published:** 2025-11-27

**Authors:** Wen Li, Junjie Shi, Parinya Lewis Tangpakonsab, Bin Zhang, Thomas Haunold, Alexander Genest, Nevzat Yigit, Leonard Atzl, Esko Kokkonen, Yong Qin, Günther Rupprechter

**Affiliations:** 1 State Key Laboratory of Coal Conversion, Institute of Coal Chemistry, Chinese Academy of Sciences, Taiyuan 030001, China; 2 Institute of Materials Chemistry, 27259 TU Wien, Vienna A-1060, Austria; 3 College of Materials Science and Engineering, Qingdao University of Science and Technology, Qingdao, Shandong 266042, China; 4 MAX IV Laboratory, 5193 Lund University, Lund SE-221 00, Sweden; 5 Center of Materials Science and Optoelectronics Engineering, University of Chinese Academy of Sciences, Beijing 100049, China

**Keywords:** CeO_2_, methane to methanol, *in situ* AP-XPS, *in situ* DRIFTS, DFT, reaction mechanism

## Abstract

The direct conversion of methane to methanol (DCMM) under continuous flow and atmospheric pressure offers notable environmental benefits and industrial promise, but remains a long-standing challenge due to the difficulty of activating CH_4_ while avoiding overoxidation of methanol. Here, we demonstrate that pure ceria (CeO_2_), without any metal promoters, enables gas-phase DCMM with up to 80% selectivity at 300–350 °C, upon optimization of the H_2_O/O_2_ ratio. At 550 °C, methanol and formaldehyde are formed at rates of 24 and 38 μmol g^–1^ h^–1^, respectively, both dropping below 1 μmol g^–1^ h^–1^ in the absence of O_2_. *Ex situ* transmission electron microscopy, X-ray photoelectron spectroscopy, and Raman spectroscopy confirm that CeO_2_ maintains structural integrity and resists carbon deposition during reaction. Combining kinetic studies, steady-state *in situ* diffuse reflectance infrared Fourier transform spectroscopy (*in situ* DRIFTS), and density functional theory (DFT) reveals that hydroxyl groups (OH), generated from water dissociation, play a multifaceted role: they facilitate C–H bond activation, promote methoxy formation, and enhance methanol desorption. *In situ* ambient-pressure X-ray photoelectron spectroscopy (AP-XPS) directly reveals the evolution of surface intermediates and shows that cofeeding O_2_ and H_2_O suppresses CH_3_O and CH_*x*_ accumulation while boosting methanol yield, indicating a rapid intermediate turnover as key to sustained activity. AP-XPS O 1s spectra further highlight that O_2_ promotes H_2_O dissociation, regenerating reactive OH groups and maintaining performance at elevated temperature. These findings offer molecular-level insights into how water and oxygen cooperatively tune reactivity, enabling efficient methane-to-methanol conversion on a metal-free oxide catalyst.

## Graphical abstract

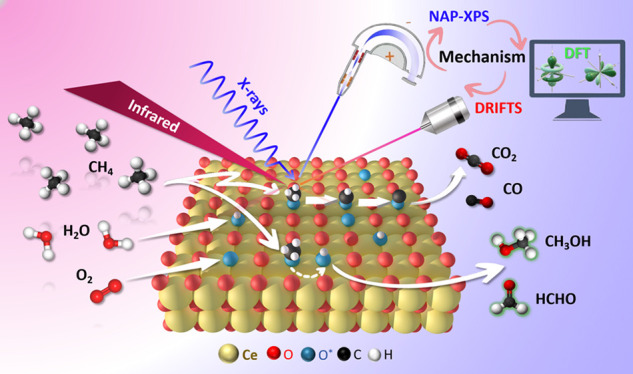

## 1 Introduction

The surge in shale gas production over recent decades has led to an abundant and low-cost supply of methane (CH_4_), making it an attractive feedstock for energy generation and chemical manufacturing. Among its potential products, methanol (CH_3_OH) is particularly valuable due to its ease of storage, transport, and role as a platform molecule for producing olefins, aromatics, and other bulk chemicals.^1–8^ However, the current industrial route to methanol relies on a high-temperature (∼1000 °C), high-pressure (30 bar) two-step process involving CH_4_ reforming to syngas followed by methanol synthesis.^2,7,9,10^ Developing a low-temperature, direct conversion of methane to methanol (DCMM) under atmospheric pressure thus represents a major opportunity to reduce the energy required and streamline CH_4_ utilization. Despite its appeal, DCMM remains a formidable challenge. The high bond dissociation energy of CH_4_ (439 kJ mol^–1^), coupled with the thermodynamic tendency of CH_3_OH to undergo overoxidation to CO and CO_2_, creates a narrow kinetic window for selective activation.^2,3,11^

Research over the past decades has focused on two major strategies: liquid-phase oxidation—typically requiring high pressures or costly oxidants such as H_2_O_2_ ^4,5,12^ —and gas-phase routes using oxidants like O_2_, N_2_O, or H_2_O under chemical looping or continuous-flow conditions.^1,13,14^ While the liquid-phase route often delivers higher CH_3_OH yields (e.g., 1300 μmol g_cat_^–1^ h^–1^ on Au/H-MOR at 150 °C),^15^ continuous-flow gas-phase systems are more amenable to scale-up and long-term operation. Yet, gas-phase CH_3_OH productivity remains modest, often limited by short gas–solid contact times and catalyst deactivation.^14^ From an industrial perspective, however, continuous gas-phase processes may prove more economical over time, as they avoid the need for periodic reactant addition and catalyst replacement inherent to batch systems. Consequently, the development of more efficient catalysts or processes tailored for continuous-flow reactors remains a key objective.

Inspired by the activity of methane monooxygenase (MMO) enzymes, catalyst design has been centered on metal-containing zeolites (e.g., Cu-, Fe-, Rh-based SSZ-13, ZSM-5, MOR).^2,4,5,12,14^ More recently, ambient-pressure X-ray photoelectron spectroscopy (AP-XPS) and ultrahigh vacuum (UHV) studies have revealed that certain oxide surfaces—including IrO_2_ (110) and CeO_*x*_/Cu_2_O/Cu­(111)—can activate CH_4_ at surprisingly low temperatures, even below room temperature.^3,11,16–18^ In particular, cofeeding CH_4_, O_2_, and H_2_O over CeO_*x*_/Cu_2_O/Cu­(111) has been shown to yield CH_3_OH selectively (∼70%) at 170 °C.^3^ However, these model studies are typically carried out under idealized UHV conditions, raising questions about their relevance to practical, high-pressure or atmospheric catalytic systems—commonly referred to as the “pressure gap” and “materials gap.”

To bridge these gaps, we investigated the direct conversion of methane to methanol over ceria-based catalysts under continuous-flow, atmospheric-pressure conditions. Remarkably, we found that even pure CeO_2_—without the addition of noble or transition metals—catalyzes DCMM with high selectivity (∼80%) at 350 °C. While higher temperatures enhance productivity (up to 24 μmol g_cat_^–1^ h^–1^ CH_3_OH at 550 °C), they also lead to overoxidation. Notably, conventional metal loading methods (e.g., Au or Pt deposition) on ceria decreased methanol selectivity and increased CO_2_ formation, contrary to trends observed in metal/oxide model systems (Figure S1).^19^

To uncover mechanistic origins of these effects, we combined steady-state catalytic evaluation with *in situ* diffuse reflectance infrared Fourier transform spectroscopy (DRIFTS), ambient-pressure XPS, and DFT calculations. Our results show that lattice oxygen initiates CH_4_ activation, while surface OH—replenished by H_2_O and stabilized by O_2_—facilitates both C–H bond cleavage and methanol desorption. This cooperative interplay between water and oxygen not only sustains high activity but also suppresses overoxidation by balancing the formation and removal of surface intermediates. These findings establish a unified mechanistic picture for DCMM over metal-free CeO_2_, offering design principles for selective methane valorization under industrially relevant conditions.

## 2 Experimental Section

### 2.1 Synthesis of CeO_2_ Nanorods

All chemicals were of analytical grade and thus used without further purification. CeO_2_ nanorods were synthesized via a hydrothermal method previously reported: 9 mmol of Ce­(NO_3_)_3_·6H_2_O (purity 99.5%, Shanghai Macklin Biochemical Technology Co.) was dissolved in 20 mL of deionized water and added dropwise to NaOH solution (6 M; purity 99.5%, Guoyao company) while stirring at room temperature for 30 min.^20^ The mixture was transferred to a 200 mL polytetrafluoroethylene (PTFE) lined stainless-steel autoclave and kept at 100 °C for 24 h. To eliminate the influence of residual Na^+^ ions, the precipitate was centrifuged and washed thoroughly with deionized water (∼15 cycles) until the pH of supernatant was neutral (pH = 7). Inductively Coupled Plasma Optical Emission Spectroscopy (ICP-OES) analysis confirmed that the Na^+^ content in the catalyst was 0.1 wt %, indicating effective removal of residual sodium via thorough washing. Moreover, no Cl^–^ containing precursors were used during synthesis, and ultrapure water with a resistivity of 18.2 MΩ·cm was employed. Thus, the negligible levels of Na^+^ and Cl^–^ exclude any significant impact on the experimental results. The precipitation was dried at 80 °C overnight and then calcined in a muffle furnace at 400 °C for 4 h (ramping rate of 4 °C/min).

### 2.2 Catalytic Performance Tests

The catalytic performance of catalysts was tested in a fixed-bed flow reactor under atmospheric pressure. The reactor was a straight quartz tube with an inner diameter of 10 mm. A thermocouple was placed inside the reactor tube to monitor the temperature of the catalyst bed. The powder catalysts were placed in between two quartz wool plugs in the constant temperature section of the reactor, the spaces above and below the quartz wool were filled with 16–30 mesh quartz sand. The catalyst was pretreated in O_2_ atmosphere for 2 h at 450 °C before the reaction. For the DCMM measurements, the reaction feed consisted of a mixture of CH_4_ (29 vol %, 285.1 mbar), O_2_ (9 vol %, 95.0 mbar), H_2_O (53 vol %, 538.1 mbar), balanced with N_2_, a total flow of 160 mL min^–1^. The gas composition was adjusted by mass flow controllers (Seven-star, China). Water (vapor) was introduced into the reactor by a syringe pump at a rate of 0.05 mL min^–1^, passing a vaporization chamber (200 °C) before entering the tube reactor. After reaction, the gas mixture first passed a circulation condenser cooled to 3 °C, with the liquid collected every 30 min. The line temperature between the reactor and the condenser were maintained at ∼ 100 °C to prevent condensation and ensure complete collection of liquid products. In this way, the product (mainly methanol) was condensed and dissolved in the liquid H_2_O, with almost no methanol remaining in the gas phase. According to Raoult’s law, in a CH_3_OH-H_2_O mixture (*X*_*CH*_3_ *OH*_ = 2 × 10^–5^, 3 °C) the gas phase methanol content is around 0.95 ppm, which is negligible. Both the liquid and gas feed components and products were analyzed by an online Fuli-F80 gas chromatograph (GC), which was equipped with a thermal conductivity detector (TCD) and two flame ionization detectors (FID). The gas composition was analyzed by Porpark Q and TDX-01 packed columns combined with the TCD. The liquid composition was analyzed by a capillary column RB-5 combined with the FID. The detection limits for CO/CO_2_ and CH_3_OH were around 100 and 1 ppm, respectively.

To further confirm that the liquid sample contains products like CH_3_COOH, CH_3_OOH, HCOOH, HCHO, which are difficult to identify by GC, ^1^H NMR spectroscopy analysis was applied. Typically, 0.2 mL of the obtained liquid product solution was mixed with 0.4 mL D_2_O (as internal standard), with the analysis conducted on an AVANCE 400 MHz UnityPlus spectrometer (Bruker). The water suppression ^1^H NMR spectroscopy was recorded by the ″noesygppr1d″ pulse sequence with acquisition parameters of fid (TD) size 32k, scan number (NS) 8, and acquisition time 2.56 s. The chemical shifts of CH_3_OOH, CH_3_OH, HCOOH, and CH_3_COOH were 3.7, 3.2, 8.2, and 1.9 ppm, respectively.

Notably, due to the low detection limit for methanol and low content of methanol in the product, errors induced by the environment or operation need to be eliminated. It was first made sure that the deionized water (reactant) did not contain CH_3_OH or any other chemicals. To prevent contamination or carryover between runs, the system was thoroughly cleaned before each run, with cleanliness confirmed by GC analysis of the rinsing solution. To eliminate interference between different samplings, the automatic inlet needle was washed five times with deionized water before and after each sampling. In between the measurements, deionized water was also intermittently used to avoid residual methanol. All samples were measured on the same day of the catalytic test to reduce errors caused by evaporation. For each catalyst, at least two intermittent catalytic cycles (250–550 °C) and three repeated experiments were carried out to ensure data reproducibility. At each temperature, at least four samples (around 2 h) were collected to determine the corresponding CH_3_OH yields.

Kinetic experiments were conducted under the same conditions as described above, as the CH_4_ conversion in the whole temperature range (300–500 °C) was far below 15%. Meanwhile, calculations based on the Weisz–Prater and Mears criteria^21^ indicate that the ceria-catalyzed DCMM reaction is not affected by mass or heat transfer limitations and is therefore kinetically controlled (S1). The reaction orders of CH_4_ and O_2_ in the DCMM reaction were determined at 500 °C, by varying the ratios of the gas-phase reactants. Long-term stability tests were performed at 450 °C under the same conditions. The conversion of CH_4_ to CH_3_OH/CO_2_/CO was quantified by the area normalization method. The conversion and selectivity were calculated by the following equations:

Methanol rate:

$$Y=\frac{C_{m}\times V}{M\times m_{cat}\times t}$$ 1

where *Y* is the methanol rate (μmol g^–1^ h^–1^), *C*_*m*_ is the concentration of methanol (g mL^–1^) measured by gas chromatography, *V* is the volume of the liquid phase product (mL) collected within a certain reaction time, *M* is the relative molecular mass of methanol (32 g mol^–1^), *m*_*cat*_ is the amount of catalyst (g), and *t* is the reaction time (h).

Formaldehyde concentration:

$$C_{f}=\frac{A_{s}-A_{b}}{k}$$ 2

Where *C*_*f*_ is the formaldehyde concentration (mM), *A*_*s*_ is the absorbance of the sample solution, *A*_*b*_ is the absorbance of the blank solution, *k* is the slope of the standard curve.

Calibration curves for methanol and formaldehyde are shown in Figure S2a.

Conversion of product *i* (CH_3_OH/CO_2_/CO/HCHO):

$$X_{i}=\frac{n_{i}}{n_{{CH}_{4},0}}$$ 3

where *X*_*i*_ is the conversion of product *i*, *n*_*i*_ is the flow rate of product *i* (mol min^–1^) under steady state, and *n*_*CH*_4_,0_ is the flow rate of CH_4_ (mol min^–1^) from the reactor inlet.

Total Conversion of CH_4_:

$$X_{total}=X_{{CH}_{3}OH}+X_{{CO}_{2}}+X_{CO}+X_{other}$$ 4

Where *X*_*total*_ is the total conversion of CH_4_ in the DCMM reaction, *X*_*CH*_3_ *OH*_ is the conversion to CH_3_OH, *X*_*CO*_2__ is the conversion to CO_2_, *X*_*CO*_ is the conversion to CO, and *X*_*other*_ is the conversion of CH_4_ to other byproducts (HCHO).

Selectivity:

$$S_{i}=\frac{n_{i}}{\overset{N}{\underset{i=1}{\sum }}n_{i}}$$ 5

where *S*_*i*_ is the product selectivity, *n*_*i*_ is the molar amount of product *i* (mol min^–1^), and ∑*n*_*i*_ is the total molar amount of all (by-)­products (mol min^–1^).

Contact time:

$$t=\frac{L\times \pi \times r^{2}}{v_{total}}$$ 6

where *t* is the contact time (min), *L* is the catalyst bed height (1, 2, or 5 mm), *r* is the inner radius of the quartz tube (5 mm), and *v*_*total*_ is the total flow rate includes all (by-) products (mL min^–1^).

The acetylacetone colorimetric method was used to determine the formaldehyde content in the liquid products. Specifically, 3 mL of the liquid product was mixed with 2 mL of 0.25% (v/v) acetylacetone solution (the synthesis method is shown below) and heated in a water bath at 80 °C for 3 min. After cooling to room temperature, the UV–vis absorption at 413 nm was measured. The acetylacetone colorimetric method is based on the reaction between formaldehyde, acetylacetone and ammonium to form a yellow green diacetyldihydrolutidine compound, which can be measured by using a UV–vis spectrophotometry at 413 nm. By comparing the results with a plot of HCHO-acetylacetone standard solutions, the formaldehyde content was quantified (Figure S3, color reactions of different samples).

The 0.25% (v/v) acetylacetone solution was prepared by dissolving 25 g of ammonium acetate in 10 mL of water, followed by the sequential addition of 3 mL of acetic acid and 0.25 mL of acetylacetone. The mixture was diluted to 100 mL with deionized water, adjusted to a pH of 6, and stored at 2–5 °C, remaining stable for up to one month.

### 2.3 Catalyst Characterization

Transmission Electron Microscopy (TEM) and high-resolution TEM (HRTEM) were performed on a JEM-2100F electron microscope (JEOL, Japan), operated at an acceleration voltage of 200 kV.

X-ray Diffraction (XRD) measurements were performed on a Bruker D8 Advance diffractometer with a Cu–Kα monochromatic X-ray source (λ = 1.5418 Å) at 40 kV and 40 mA. The scan range was 5 ∼ 90° with a rate of 4 °/min.

X-ray photoelectron spectroscopy (XPS) was conducted on a Thermo electron spectrometer using a monochromatized Al–Kα (Thermo Scientific ESCALAB 250Xi) radiation source and a 165 mm mean radius hemispherical analyzer (overall yielding a resolution of 0.1 eV; with binding energies calibrated via the C 1s peak at 284.8 eV). XPS measurements were acquired at room temperature without sample pretreatment. Spectral analysis was carried out using Avantage software.

Low-temperature N_2_ physical adsorption–desorption measurements were conducted on an automatic Micromeritics ASAP 2460 instrument. Each sample was first degassed at 200 °C for 3 h under vacuum. The specific surface area, pore volume, and average pore size were calculated based on the Brunauer-Emmet-Teller (BET) and Barret-Joyner-Halenda (BJH) methods.

Raman spectra were recorded on a Horiba LabRAM HR Evolution spectrometer using a 532 nm laser under ambient conditions, for a scanning range of 100–1800 cm^–1^ and with a resolution of 0.5 cm^–1^.

*In situ* diffuse reflectance infrared Fourier transform spectroscopy (*In situ* DRIFTS) was carried out on a Bruker Tensor II FTIR spectrometer equipped with a liquid nitrogen-cooled mercury–cadmium–telluride (MCT) detector. The *in situ* infrared test is conducted at atmospheric pressure, matching the catalytic test conditions. For each test, around 50 mg of catalyst powder was pressed to a pellet and placed in the DRIFTS cell (PIKE Technologies DiffusIR). The catalysts were first pretreated in synthetic air (20 mL min^–1^) at 400 °C for 1 h, before cooling to 150 °C and purging with Ar (10 mL min^–1^) for 10 min. Background spectra of the sample were collected in Ar flow (10 mL min^–1^) at 150 °C. A H_2_O saturator was used to deliver water to the sample and was kept at ∼ 60 °C with 10 mL min^–1^ Ar bubbling through it. Different reaction gases (CH_4_, O_2_, N_2_, H_2_O) were introduced into the IR cell with a total gas flow rate of 30 mL min^–1^. The infrared spectra were measured at different temperatures (150, 200, 250, 300, 350, and 400 °C), with each temperature held for 0.5 h to ensure a steady state. All spectra (100 scans) were collected from 900 to 4000 cm^–1^ at a resolution of 4 cm^–1^. All reported IR spectra are difference spectra referenced to a background spectrum collected at 150 °C after pretreatment but before introducing the feed gas.

Ambient Pressure X-ray Photoelectron Spectroscopy (AP-XPS) measurements were performed at the SPECIES beamline of the MAX IV Laboratory (proposal ID: 20241326).^22,23^ AP-XPS was performed in the pressure range of 0.3–1 mbar, introducing a ∼ 3-order pressure gap relative to the catalytic process. The CeO_2_ catalyst, synthesized in-house (details see above) as a powder, was pressed into round tablets using a mold and tablet press, then mounted on the sample holder. A type K thermocouple placed between the sample and mounting plate was used to monitor the temperature. We evaluated possible beam-induced effects by continuously irradiating the same spot on the sample under vacuum for ∼ 15 min while repeatedly collecting O 1s and Ce 3d spectra. No significant changes in peak shape or intensity were observed, indicating negligible beam damage under our measurement conditions. Before each measurement, the catalyst was pretreated in 5 mbar O_2_ at 400 °C for 20 min to remove surface contaminants (e.g., water and adsorbed organics) and to stabilize the surface. This pretreatment was repeated between measurements (i.e., when switching feed gases) to ensure surface regeneration, as confirmed by comparison of the survey spectra. To correct for charging-induced shifts, the Ce 4d peak shift under each condition (temperature, gas composition, and photon energy) was used as an internal reference to calibrate the Ce 3d, C 1s, and O 1s spectra. During AP-XPS measurements, the CeO_2_ samples were exposed to different gas compositions (CH_4_/O_2_/H_2_O, CH_4_/O_2_, CH_4_/H_2_O, and CH_4_) across a temperature range of 250–400 °C. The C 1s, O 1s, and Ce 3d regions were probed with photon energies of 430, 670 and 1200 eV, respectively, with a total energy resolution of approximately 0.25 eV. The AP-XPS data were analyzed using XPS-peak software for peak fitting, with a Shirley background subtraction method applied to remove the baseline background signal. The peak widths were constrained between 1.0 and 2.0 eV. Peak assignments of CH_*x*_, CH_3_O, and CO_*x*_ species were primarily based on deconvolution of the C 1s spectrum and comparison with assignments reported by Rodriguez and co-workers.^24–27^ Control experiments on ceria after O_2_ pretreatment, as well as surface restoration by O_2_ treatment (6 mbar, 400 °C, 15 min) between reaction cycles, showed no detectable carbon-related peaks, confirming that the observed C species mainly originate from CH_4_ activation.

### 2.4 DFT Calculations

Spin-polarized density functional theory (DFT) calculations were carried out using the Vienna Ab initio Simulation Package (VASP)^28^ with a utilization of projector augmented-wave formalism (PAW).^29,30^ The exchange-correlation energy was parametrized using generalized gradient approximation (GGA) according to Perdew–Burke–Ernzerhof (PBE) functional.^31^ The energy cutoff for plane wave basis set was set to 450 eV with a 3 × 3 × 1 k-point sampling using a Monkhorst–Pack mesh^32^ for all calculations. The localization of 4f-orbitals of Ce atoms was treated with the Hubbard model using the Dudarev scheme^33^ where the effective U parameter was set to 5.0 eV.^34–36^ Localized sites of Ce^3+^(4f) species were controlled by an occupation matrix^37^ first before full relaxation without orbital constraint. The electronic self-consistency was set to converge when the total energy difference was lower than 10^–6^ eV. All atoms were relaxed until the forces were less than 0.02 eV/Å (0.03 eV/Å for NEB images).

Nanorods are often described as exposing {110}/{100} facets, but spectroscopic and microscopic results show that they reconstruct to expose a large number of {111} nanofacets, that remain stable even under oxidizing conditions.^38,39^ Given that (111) is thermodynamically the most stable surface and dominates the catalytic properties after reconstruction, we used this surface to model the reaction mechanism. A p(2 × 2)-CeO_2_(111) slab model with a thickness of 11.0 Å was used for surface calculations. The vdW-dispersion energies by D3 method of Grimme et al.^40^ were included to correct the total energies. A 12 Å vacuum gap and a dipole moment correction were included in the direction perpendicular to the surface. Reaction energies were calculated by *E*_r_ = *E*_pro_ – *E*_react_ where *E*_pro_ and *E*_react_ are the total energies of product and reactant states, respectively. Transition states were identified using the nudged elastic band method (NEB)^41^ and barrier heights were calculated by *E*_a_ = *E*_TS_ – *E*_react_ where *E*_TS_ and *E*_react_ are the total energies of transition and reactant states, respectively. All transition states were confirmed to have exactly one imaginary mode by vibrational analysis using a finite-difference approach with a displacement width of 0.01 Å. A charge analysis was carried out following the procedure suggested by Bader.^42,43^

## 3 Results and Discussion

X-ray diffraction (XRD) confirms that the synthesized CeO_2_ adopts a cubic fluorite structure (PDF#34–0394; Figure S4). XPS analysis of CeO_2_ by deconvolution of the Ce 3d spectrum yields a surface Ce^3+^ content of ∼20.0%, with each Ce^3+^ associated with one oxygen vacancy.^44–46^ Similarly, deconvolution of the O 1s spectrum gives an OH peak at ∼531.4 eV, commonly attributed to water dissociation at oxygen vacancies, corresponding to ∼13.7%. Hence, the oxygen vacancy concentration in the ceria rods is estimated to be 14–20% (Figure S5). TEM and HRTEM images reveal well-defined nanorods with an average diameter of ∼10 nm and length of ∼100 nm (Figure 1a, b). After 10 h of reaction between 300–550 °C, the rod-like morphology remains largely unchanged (Figure 1c, d). BET analysis shows that the specific surface area decreases from ∼91.3 to 52.7 m^2^ g^–1^ (Figure S6). However, N_2_ adsorption isotherms before and after reaction confirm that the samples retain mesoporous structures without micropores. Together with XRD results (Figure S4), which show an increase in mean crystallite size from 12.2 to 14.4 nm, these findings indicate that the surface area loss mainly arises from sintering during reaction. Thus, CeO_2_ nanorods largely retain their morphology and crystallinity, with only moderate textural degradation.

**Figure 1:**
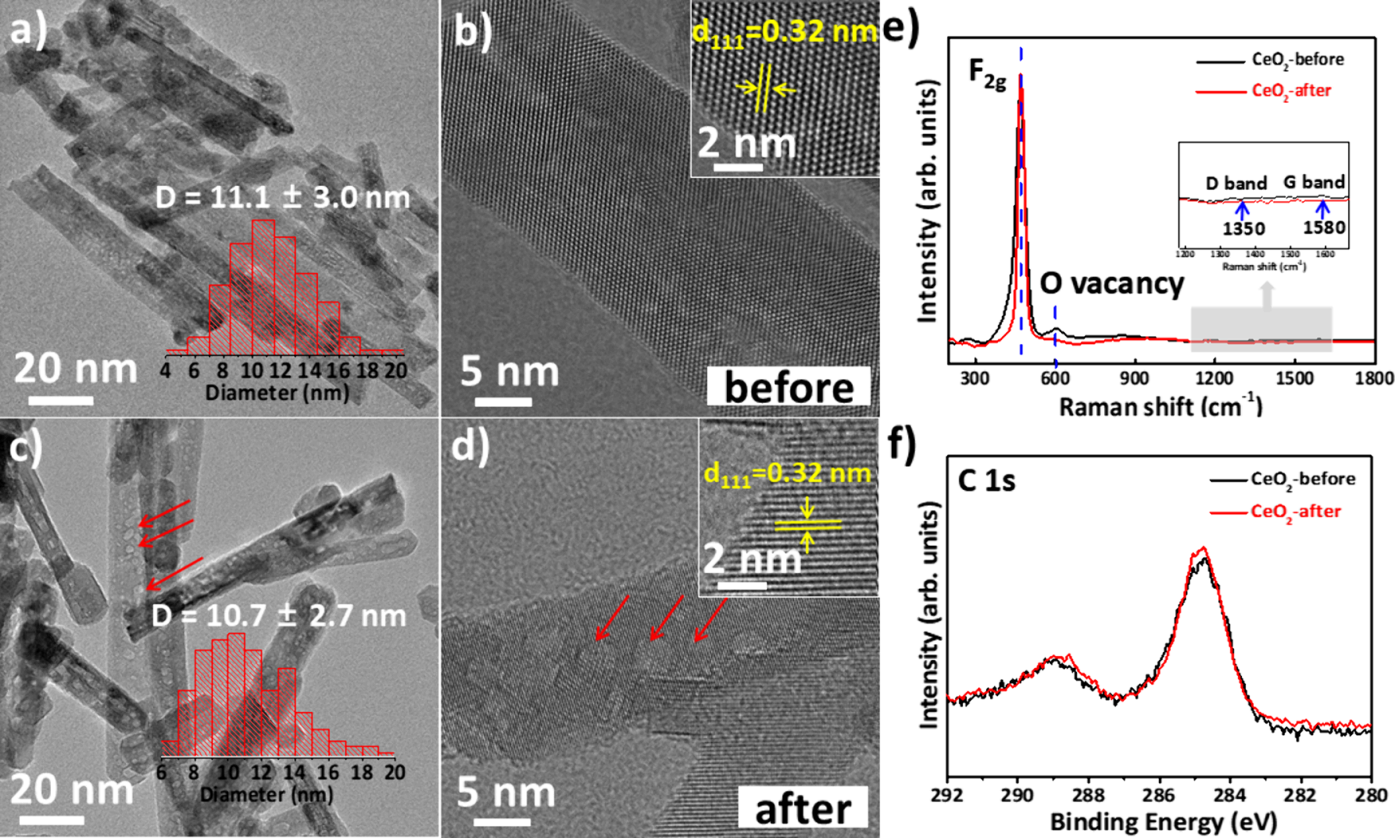
Morphology and surface species study of CeO_2_ before and after reaction. TEM images of CeO_2_ nanorods (a) before and (c) after catalyzing the DCMM reaction (inset: rod diameter distribution of CeO_2_). HRTEM images of CeO_2_ (b) before and (d) after catalyzing the DCMM reaction (inset: lattice planes of CeO_2_). Red arrows in panels (c) and (d) indicate voids formed on the ceria after the reaction. (e) Raman and (f) photoemission C 1s spectra of CeO_2_, before and after the DCMM reaction.

After reaction, no carbon deposition was observed on CeO_2_ as evidenced by TEM analysis (absence of filamentous carbon species) (Figure 1c)^47^ and supported by XPS C 1s spectra, which show negligible changes in carbon-related peaks before and after DCMM (Figure 1f). Raman spectroscopy further confirms the absence of graphitic species, with no significant variation in the D (1350 cm^–1^) and G (1580 cm^–1^) bands (Figure 1e).^48^ These results, along with the unchanged catalyst color (Figure S7), suggest that CeO_2_ exhibits excellent resistance to coking under reaction conditions.^49–51^ High-resolution TEM reveals lattice fringes corresponding to the (111) and (220) planes (0.32 and 0.19 nm, respectively; Figure 1b, d insets),^20,52–54^ confirming the structural integrity of the fluorite phase. However, after reaction, noticeable surface pits and depressions emerge (Figure 1d), originating from precursor decomposition during cerium oxide formation, and becoming more pronounced after reaction, likely due to thermal treatment.^55^ This morphological evolution coincides with a decrease in oxygen vacancy Raman signals (Figure 1e), suggesting that redox-active sites undergo restructuring during catalysis.

Control experiments confirmed that thermal activation alone does not account for methanol formation: no CH_3_OH was detected below 450 °C in the absence of catalyst, and only trace amounts (∼0.3 μmol h^–1^) appeared at 500 °C, likely due to wall effects from the quartz reactor (Figure S9). Figure 2a shows that increasing the catalyst mass from 100 to 500 mg—thereby reducing the gas hourly space velocity (GHSV) from 96,000 to 19,200 mL g^–1^ h^–1^—led to a modest increase in CH_4_ conversion (from 3.8 × 10^–4^% to 3.4 × 10^–2^%) but a pronounced drop in methanol yield (from 3.3 to 0.9 μmol g^–1^ h^–1^) at 350 °C. This decline is attributed to longer gas–solid contact times (0.03 to 0.15 s), which promote overoxidation to CO_2_, consistent with prior reports by Somorjai et al. on molybdena-based catalysts.^56^ To minimize overoxidation and maximize methanol selectivity, a contact time of 0.03 s (*m*_*catal*_ = 100 mg) was employed for all subsequent experiments.

**Figure 2:**
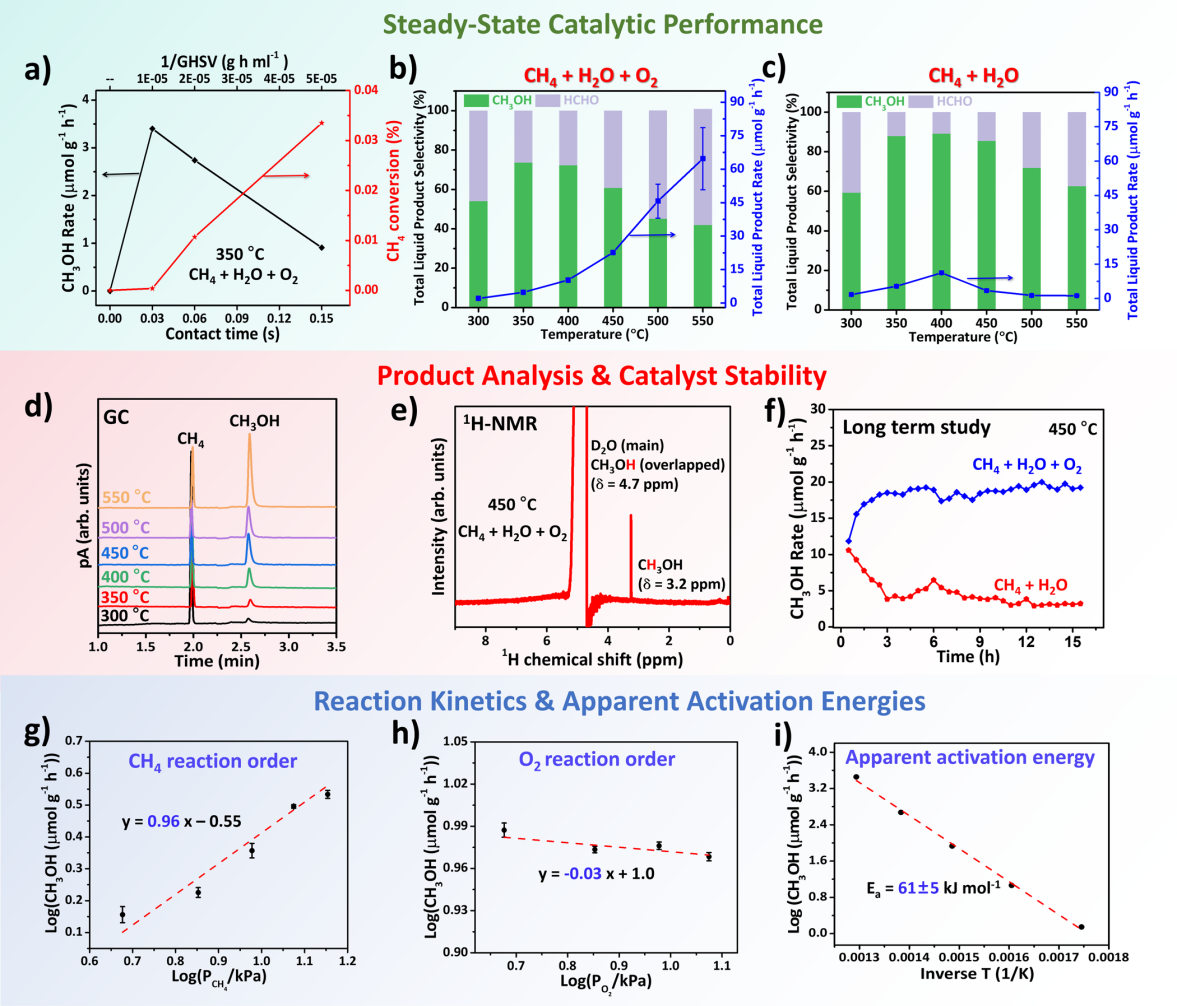
Steady-state catalytic performance, product distribution, stability, and reaction kinetics. (a) CH_3_OH formation rates and CH_4_ conversion as a function of contact time (or space velocity) at 350 °C (Table S1). Reaction conditions: CH_4_ (29 vol %, 285.1 mbar), O_2_ (9 vol %, 95.0 mbar), H_2_O (53 vol %, 538.1 mbar), N_2_ (9 vol %, 95.0 mbar), total flow rate: 160 mL min^–1^. (b,c) Temperature-dependent selectivity toward CH_3_OH and HCHO, along with the total liquid product formation rate, under reaction conditions of (b) CH_4_ + H_2_O + O_2_ (29:53:9 vol %) and (c) CH_4_ + H_2_O (29:53 vol %). Conditions: total flow = 160 mL min^–1^, catalyst mass = 100 mg, space velocity = 96,000 mL g^–1^ h^–1^. (d) GC profiles of liquid products, showing CH_4_ and CH_3_OH peaks at ∼2.1 and ∼2.7 min, respectively. (e) ^1^H NMR spectrum of liquid products collected over CeO_2_ after direct methane-to-methanol conversion at 450 °C. (f) Long-term stability test of CeO_2_ in CH_4_ + H_2_O + O_2_ and CH_4_ + H_2_O feeds at 450 °C for 15 h on stream. (g, h) Double-logarithmic plots for determination of reaction orders with respect to (g) CH_4_ and (h) O_2_. Conditions: Temperature = 500 °C, catalyst mass = 200 mg, H_2_O = 0.05 mL min^–1^, space velocity = 48,000 mL g^–1^ h^–1^. In (g), O_2_ flow fixed at 45 mL min^–1^ while CH_4_ varied from 7.5 to 22.5 mL min^–1^ (47–142 mbar); in (h), CH_4_ flow fixed at 45 mL min^–1^ while O_2_ varied from 7.5 to 18.75 mL min^–1^ (47–119 mbar). The reaction order of O_2_ at lower pressure (5–24 mbar) was also tested, as shown in Figure S8. (i) Apparent activation energy of CeO_2_ calculated from Arrhenius plot in the temperature range of 300–500 °C under standard feed (CH_4_ 29 vol %, O_2_ 9 vol %, H_2_O 53 vol %, N_2_ 9 vol %, 160 mL min^–1^).

CeO_2_ catalyzes DCMM over a wide temperature range (300–550 °C), with both methanol and formaldehyde detected as low as 300 °C (Figure 2b). A carbon balance within ±3% was achieved under the applied reaction conditions (Table S2). As temperature increased, product yields rose—peaking at 550 °C with 24 μmol g^–1^ h^–1^ methanol and 38 μmol g^–1^ h^–1^ formaldehyde—but selectivity dropped sharply due to overoxidation. At 350 °C, methanol accounted for 80% of carbon-containing products, while at ≥500 °C, CO_2_ became dominant (∼99%), accompanied by minor CO and trace oxygenates (Figure S10a). The long-term stability tests at 350 °C show that the methanol rate (∼3.3 μmol g^–1^ h^–1^) and selectivity (∼85%) remains almost unchanged over 55 h (Figure S11), indicating that the catalyst exhibits good stability. Still, the methanol yield at 350 °C (∼3.3 μmol g^–1^ h^–1^) was 12.7 times higher than that reported by Zhang et al. over 5.5 nm-CeO_2_ nanoparticles (0.26 μmol g^–1^ h^–1^ at 180 °C) and comparable with the one reported by Koishybay et al. over Cu-SSZ-13 (5.30 μmol g^–1^ h^–1^ at 225 °C).^57,58^ These trends highlight the classic activity–selectivity trade-off in methane oxidation. Notably, gas-phase byproducts are limited to CO and CO_2_, with no CH_3_OOH, HCOOH, or CH_3_COOH detected in the liquid phase by ^1^H NMR (Figure 2e). This suggests that CeO_2_ favors partial oxidation to methanol/formaldehyde over deep oxidation under controlled contact times.

To assess whether H_2_O alone can support DCMM, the feed was modified to include only CH_4_ and H_2_O. As shown in Figure 2c, methanol yields at ≤400 °C were comparable to those obtained with CH_4_ + H_2_O + O_2_, indicating that water can serve as an effective oxidant under mild conditions. However, at higher temperatures, CH_3_OH and HCHO yields declined significantly, while CO_2_ became the dominant product. Selectivity dropped from ∼90% at 350 °C to <2% at 550 °C, suggesting that methanol undergoes reforming or overoxidation at elevated temperatures (Figure S10b). This behavior likely arises from competing reforming reactions: methanol reforming (CH_3_OH + H_2_O → CO_2_ + 3H_2_) and steam reforming of methane (CH_4_ + H_2_O → CO + 3H_2_ followed by water–gas shift reaction), both of which are thermodynamically favored at high temperatures. In contrast, when O_2_ is included in the feed (CH_4_ + H_2_O + O_2_), the methanol yield continues to increase with temperature, indicating that O_2_ suppresses reforming and stabilizes the surface for selective oxidation.

Catalyst stability was evaluated under continuous operation using different feed compositions (Figure 2f). With CH_4_ + H_2_O + O_2_ at 450 °C, methanol yields remained stable (∼19 μmol g^–1^ h^–1^) over 15 h. In contrast, under CH_4_ + H_2_O only, the yield declined rapidly from ∼10 to ∼4 μmol g^–1^ h^–1^ within the first 2 h, then maintained at ∼3–4 μmol g^–1^ h^–1^. This decline is not attributed to carbon deposition (different from reports by Lustemberg et al. over Ni/CeO_2_ catalyst)^59^—as XPS C 1s spectra show negligible changes (Figure S12)—but to gradual depletion of surface-active oxygen species in the absence of O_2_. Reintroduction of O_2_ to the CH_4_ + H_2_O feed at 450 °C led to immediate restoration of methanol productivity (Figure S13), confirming that continuous oxygen replenishment is essential for sustained activity. These results highlight that while H_2_O alone can *transiently* support DCMM at low temperatures, long-term selectivity and yield require the cooperative role of both H_2_O and O_2_.

To probe the role of surface oxygen species, hydrogen was introduced as a competing reductant in the CH_4_ + H_2_O feed. As shown in Figure S14, the addition of H_2_ significantly suppressed methanol formation—yielding <1.2 μmol g^–1^ h^–1^ at ≤400 °C and virtually no methanol at 450–500 °C. This suggests that H_2_ either consumes or blocks reactive surface oxygen species, thereby hindering C–H bond activation. Upon cofeeding O_2_ (CH_4_ + H_2_O + H_2_+ O_2_), methanol yields were restored (Figure S14, red line). Below 400 °C, performance mirrored that of CH_4_ + H_2_O; above 400 °C, the yield increased substantially, reaching 14 μmol g^–1^ h^–1^ at 450 °C—exceeding that of CH_4_ + H_2_O alone. These results highlight the critical role of molecular O_2_ in replenishing active oxygen species on CeO_2_, particularly under reductive or high-temperature conditions where surface O becomes depleted. Without this replenishment, the DCMM cycle cannot be sustained.

To identify the rate-limiting step in DCMM over CeO_2_, kinetic studies were conducted by varying CH_4_ or O_2_ partial pressures while keeping other parameters constant (quasi-series method).^60^ As shown in the double-log plots (Figure 2 g, h), the reaction rate displayed near-first-order dependence in CH_4_ (slope = 0.96) and near-zero-order dependence in O_2_ (slope = – 0.03), indicating that methane activation is the rate-determining step, while oxygen plays a minimal role in the rate expression. This result aligns with the kinetic study by Somorjai et al., who reported a similar first-order dependence on CH_4_ and zero-order dependence on N_2_O during the partial oxidation of methane to methanol using a silica-supported molybdena catalyst.^56^ The apparent activation energy (*E*_a_) for methanol formation was determined to be ∼61 ± 5 kJ mol^–1^ in the 300–500 °C range (Figure 2i), which is significantly lower than previously reported values for Cu/SSZ-13 (∼97 kJ mol^–1^) and Mo_2_O_3_/SiO_2_ (∼171 kJ mol^–1^).^13,56^ This relatively low barrier suggests that the CeO_2_ surface offers a more favorable energetic pathway for C–H bond cleavage and product desorption, in line with experimental observations of activity at lower temperatures.

To probe the kinetic regime more precisely, the oxygen partial pressure range was extended to 5–24 mbar, approaching the edge of the zero-order region (Figure S8a). The resulting rate dependence on P_O2_ yielded a slope of – 0.025, indicating that the reaction rate remains essentially independent of oxygen pressure. This observation further supports that adsorbed oxygen species (O*) are not involved in the rate-determining step. Moreover, Arrhenius plots (Figure S8b) constructed at CH_4_: O_2_ feed ratios of 4:1 and 2:1 exhibited nearly identical activation energies (within experimental error), reinforcing the invariance of *E*_*a*_ and suggesting a consistent reaction mechanism across varying feed compositions.

Steady-state *in situ* DRIFTS was employed to monitor surface species evolution under various feed compositions (CH_4_, CH_4_ + O_2_, CH_4_ + H_2_O, CH_4_ + O_2_ + H_2_O) across 150–400 °C (Figure 3). In the OH stretching region (3750–3450 cm^–1^), all feeds exhibit negative bands whose intensity increases with temperature, indicating progressive consumption of surface hydroxyls either through desorption or their involvement in C–H bond activation.^61,62^ With water vapor present, broader OH features emerge (∼3000–3500 cm^–1^), and negative OH signals shift to higher temperatures, suggesting continuous OH replenishment via H_2_O dissociation (Figure 3c–d). Gas-phase methane-related C–H stretching bands (3240–2840 cm^–1^) dominate at all temperatures, while a distinct peak at 2840 cm^–1^—assigned to the first overtone of CH_3_ deformation in OCH_3_ species (according to Badri et al.) intensifies under CH_4_ + O_2_ (Figure 3b), consistent with enhanced methoxy formation via oxidative activation.^63^ Although this peak typically appears alongside the 2922 cm^–1^ peak (a Fermi resonance of CH_3_ vibrational modes), distinguishing it is challenging due to overlap with gas-phase methane peaks.^63^ CO_2_ (2330 cm^–1^) and carbonate-related bands (2400–2500 cm^–1^) also appear above 250 °C across all feeds.^61,64–66^ Interestingly, carbonate formation is strongly suppressed when H_2_O is present (Figure 3c–d), indicating that water mitigates deep oxidation and promotes selectivity.

**Figure 3:**
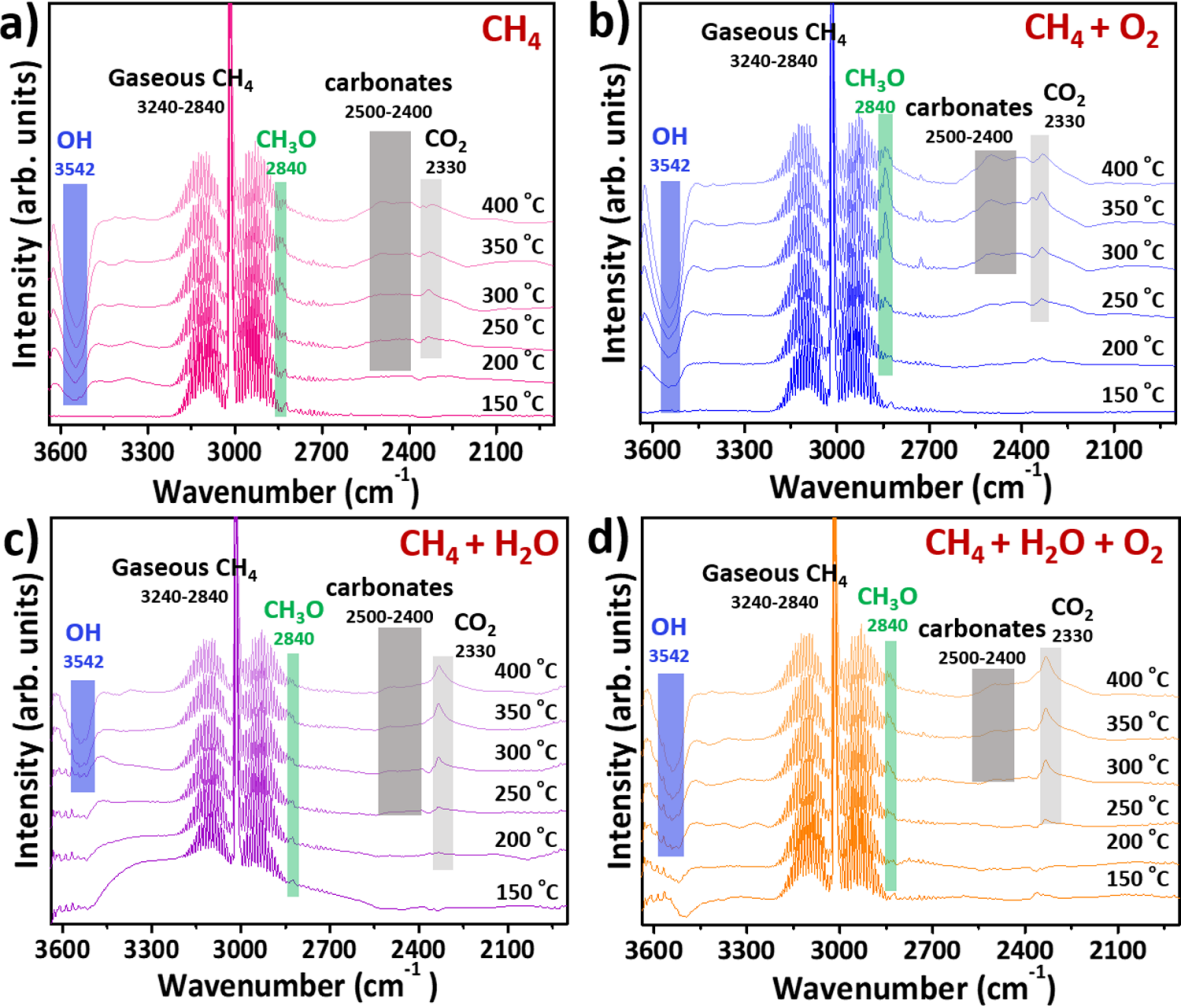
Steady-state flow *in situ* DRIFTS of CeO_2_ under various conditions (4000–1900 cm^–1^). (a) CH_4_ (28.4 vol %, 287.7 mbar), (b) CH_4_ (14.3 vol %, 145.0 mbar) + O_2_ (40.6 vol %, 411.4 mbar), (c) CH_4_ (14.3 vol %, 145.0 mbar) + H_2_O (49.5 vol %, 501.3 mbar), (d) CH_4_ (10.5 vol %, 106.3 mbar) + H_2_O (36.4 vol %, 368.9 mbar) + O_2_ (5.3 vol %, 53.7 mbar). Spectra were collected at temperatures ranging from 150 to 400 °C. In all cases, feed gases were balanced with N_2_.

The methoxy ν­(OC) stretching region (1150–950 cm^–1^) reveals three distinct bands at 1108, 1063, and 1030 cm^–1^, assigned to on-top (type I) and bridging (type II and III) methoxy species, respectively (Figure 4).^61,63,64,67–70^ Under CH_4_-only conditions (Figure 4a), type III species—bridging Ce^4+^ cations adjacent to oxygen vacancies—dominate between 200–300 °C but decline at higher temperatures, indicating desorption or transformation.

**Figure 4:**
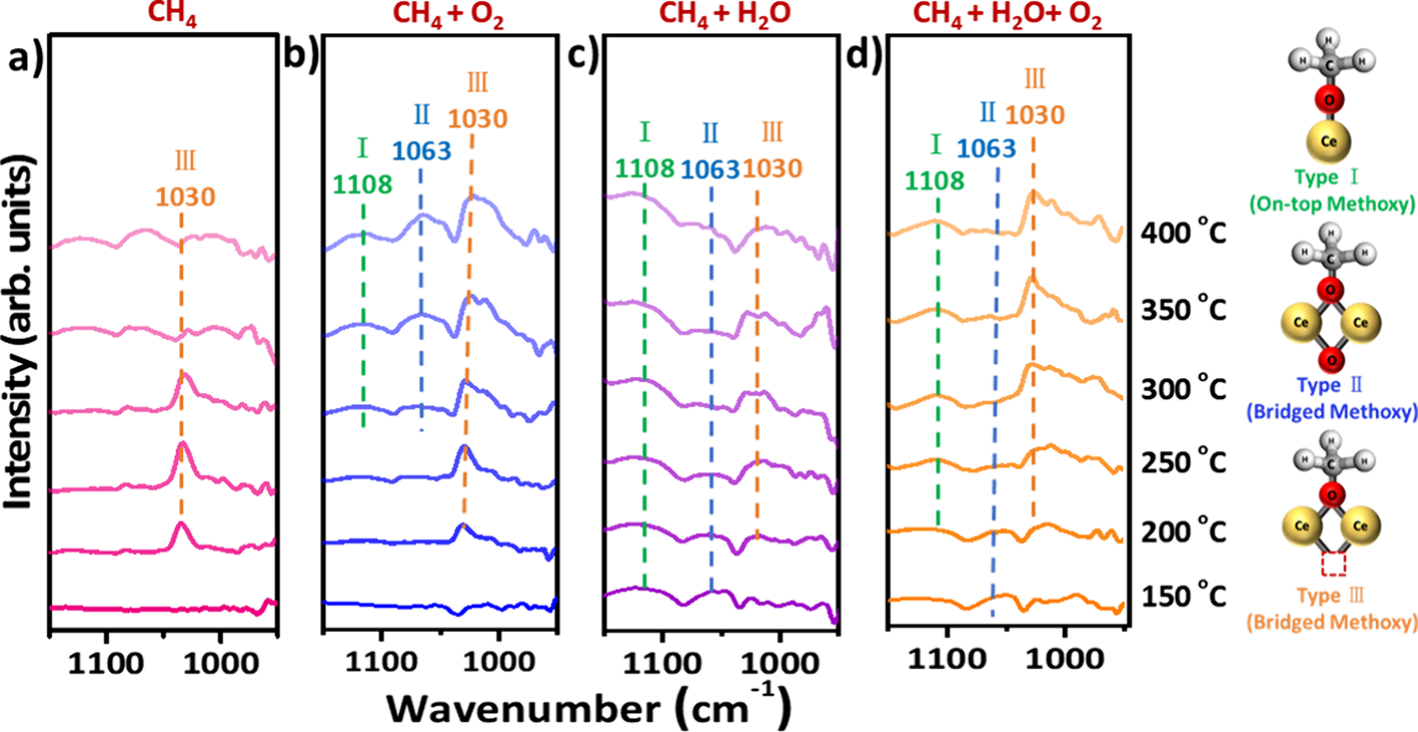
Steady-state flow *in situ* DRIFTS of CeO_2_ under various conditions (1150–950 cm^–1^). (a) CH_4_ (28.4 vol %, 287.7 mbar), (b) CH_4_ (14.3 vol %, 145.0 mbar) + O_2_ (40.6 vol %, 411.4 mbar), (c) CH_4_ (14.3 vol %, 145.0 mbar) + H_2_O (49.5 vol %, 501.3 mbar), and (d) CH_4_ (10.5 vol %, 106.3 mbar) + H_2_O (36.4 vol %, 368.9 mbar) + O_2_ (5.3 vol %, 53.7 mbar) at temperatures from 150 to 400 °C, with all feed gases are balanced with N_2_.

Adding O_2_ (Figure 4b) stabilizes all three methoxy species, particularly type III, which remains prominent even at 400 °C. This suggests that O_2_ not only sustains surface oxygen species but also promotes methoxy formation and retention under steady-state conditions. In contrast, when only H_2_O is present (Figure 4c), type III intensity is much weaker, and on-top/type II species are nearly undetectable—likely due to competitive OH adsorption and insufficient oxygen replenishment.

When both O_2_ and H_2_O are cofed (Figure 4d), type III methoxy signals increase relative to the H_2_O-only case, while type II remains less stable. This indicates that water facilitates methoxy desorption or transformation, whereas O_2_ is required to sustain the active oxygen framework for methoxy formation. The combined presence of O_2_ and H_2_O thus enables dynamic turnover of reactive intermediates—balancing stability with product release.

To gain mechanistic insights into methane activation on ceria, ambient-pressure X-ray photoelectron spectroscopy (AP-XPS; MAX IV synchrotron) was conducted at 250–400 °C under a CH_4_ feed at a pressure of 0.3 mbar (Figure 5a). In the C 1s region, spectral deconvolution reveals peaks at 284.2, 285.0, and 286.0 eV, corresponding to CH_*x*_, CH_3_O, and physisorbed CH_4_, respectively.^3,11,16,59^ A broad feature between 292–287 eV can be further resolved into contributions from CO_*x*_ species (288.5 eV) and the Ce 4s signal (290 eV), in line with previous assignments.^3,11,16,59^ At 250 °C, all major surface intermediates are already detectable. As temperature increases to 350 °C, the CH_3_O signal becomes sharper and more intense, while the CH_*x*_ intensity decreases, suggesting stronger CH_3_O stabilization and more facile CH_*x*_ desorption. Above 350 °C, all signals decline markedly, likely due to thermal desorption. These results confirm that pure CeO_2_ can activate CH_4_ to form surface-bound intermediates—a finding consistent with our *in situ* DRIFTS results but contrasting with earlier reports on CeO_*x*_/Cu_2_O/Cu­(111), where ceria alone was inactive.^3^ The discrepancy may stem from the higher reaction temperature and greater surface area of the powder catalyst used here, which likely provide enhanced exposure of redox-active oxygen species. However, under a pure CH_4_ feed, these reactive oxygen species are gradually consumed, and without replenishment, the catalytic cycle cannot be sustained—leading to deactivation at higher temperatures.

**Figure 5:**
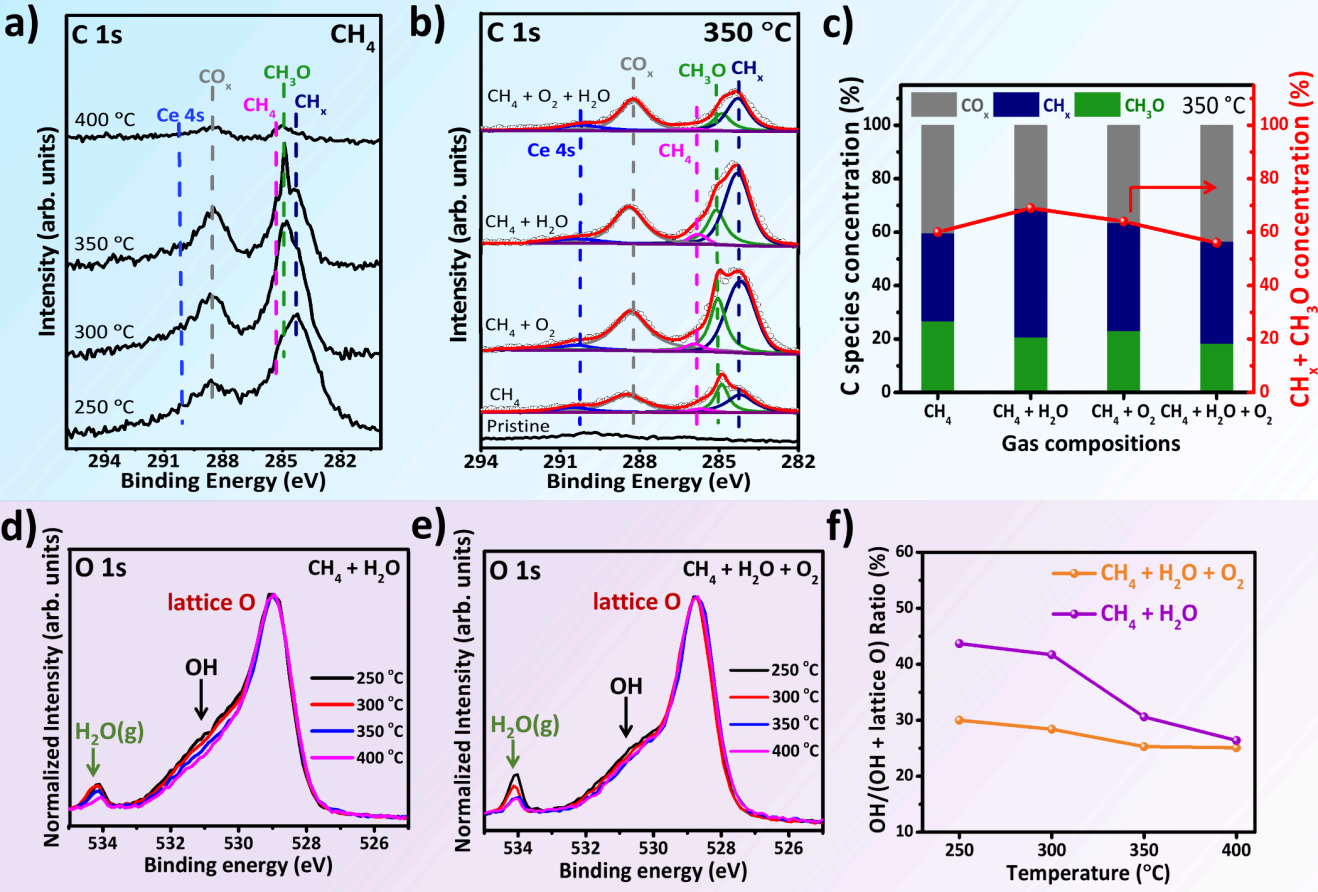
C 1s and O 1s AP-XPS spectra of CeO_2_ under CH_4_ and cofed conditions at elevated temperatures. (a) C 1s region of AP-XPS spectra showing methane interaction with CeO_2_ at temperatures ranging from 250 to 400 °C under 0.3 mbar CH_4_. (b) Comparison of C 1s spectra after exposing CeO_2_ to different gas environments at 350 °C: 0.3 mbar CH_4_; 0.3 mbar CH_4_ + 0.1 mbar O_2_; 0.3 mbar CH_4_ + 0.6 mbar H_2_O; and 0.3 mbar CH_4_ + 0.1 mbar O_2_ + 0.6 mbar H_2_O. Open circles represent raw data points; solid red lines denote the cumulative fitted peaks. (c) Relative surface concentrations of CH_*x*_, CH_3_O, and CO_*x*_ species derived from peak integration of the corresponding components in the C 1s spectra shown in (b). O 1s region of AP-XPS spectra under (d) 0.3 mbar CH_4_ + 0.6 mbar H_2_O and (e) 0.3 mbar CH_4_ + 0.1 mbar O_2_ + 0.6 mbar H_2_O, recorded at temperatures from 250 to 400 °C. The O 1s peak fitting is shown in Figure S16. (f) Temperature-dependent OH/(OH + lattice O) ratios in the two gas feeds, reflecting the evolution of surface hydroxyl species.

To address this limitation, O_2_, H_2_O, and their combination were introduced at 350 °C—a temperature threshold for CH_3_OH production under steady-state conditions (Figure 5b). The specific pressure of each gas component is indicated in the figure caption. Both O_2_ and H_2_O significantly increase the surface concentrations of CH_3_O and CH_*x*_ species. CH_4_ + O_2_ promotes CH_3_O formation, whereas CH_4_ + H_2_O leads to a stronger CH_*x*_ signal. Notably, cofeeding both O_2_ and H_2_O results in significantly lower CH_*x*_ and CH_3_O intensities than with the separate addition of either oxidant. This observation, consistent with DRIFTS results, indicates that surface intermediates are not accumulating, in line with the fact that this condition delivers the highest methanol yield and stability (Figure 2b). In contrast, prior studies on CeO_2_/Cu_2_O/Cu­(111) showed enhanced CH_3_O/CH_*x*_ coverage under the same cofeed condition.^3^ In control experiments, oxygen pretreatment (5 mbar O_2_, 400 °C, 20 min) yields a clean ceria surface, with only a small, broad Ce 4s peak and no detectable C 1s signal (Figure 5b, pristine), indicating the absence of carbon-based species.

According to the UHV model study by Mullins et al., chemisorbed methoxy groups can persist on O-vacancy-rich CeO_1.75_(111) up to ∼600 K (327 °C) based on C 1s spectra.^71^ By contrast, our DRIFTS and AP-XPS experiments were performed under steady-state conditions (1 bar or 1 mbar) with continuous reactant dosing on oxygen-defect-rich ceria powders, which may allow methoxy species to remain stable even to higher temperatures, up to ∼ 350–400 °C.

The inverse correlation between intermediate coverage and methanol productivity indicates that a high surface coverage of CH_*x*_ and CH_3_O species is not a prerequisite for high catalytic performance. Rather, an optimal balance between intermediate formation and turnover is essential for achieving high activity and selectivity. In our case, under CH_4_ + O_2_ + H_2_O conditions, enhanced oxygen mobility and efficient OH transfer promote faster conversion of surface intermediates, resulting in lower steady-state concentrations and improved methanol productivity. The suppressed accumulation may also reflect a larger fraction of surface sites actively participating in the reaction cycle rather than stabilizing intermediates. Together, these findings support a picture of a more efficient and dynamic catalytic cycle on CeO_2_ when both O_2_ and H_2_O are present, enabling higher and sustained methanol production.

Figure 5 presents normalized O 1s AP-XPS spectra collected under CH_4_ + H_2_O (Figure 5d) and CH_4_ + H_2_O + O_2_ (Figure 5e) atmospheres at various temperatures. In CH_4_ + H_2_O, the intensity of the OH-related peak (∼530.5 eV), along with the signal attributed to gas-phase H_2_O (∼534.0 eV), decreases significantly as temperature increases.^3,16,20,72^ This trend indicates that surface OH species are progressively consumed or desorbed and cannot be effectively regenerated by water alone under these conditions. In contrast, cofeeding O_2_ with H_2_O stabilizes the OH signal across the full temperature range, suggesting that O_2_ facilitates water dissociation and promotes the continuous regeneration of surface hydroxyls. This sustained OH availability is crucial for maintaining catalytic activity at elevated temperatures, as hydroxyls are directly involved in both methane activation and product formation. DFT calculations (discussed below) further support this conclusion, showing that surface OH substantially lowers the barrier for C–H bond cleavage and assists in methanol (CH_3_OH) formation. The copresence of O_2_ and H_2_O not only replenishes lattice oxygen vacancies but also ensures dynamic OH regeneration, enabling a robust catalytic cycle. This synergistic effect explains the enhanced and stable methanol productivity observed under CH_4_ + H_2_O + O_2_ conditions. Note that although the CO_3_ peak coincides with the OH signal, its contribution is minimal (∼1/20, from C 1s analysis) and is not considered here.

Moreover, our AP-XPS/DRIFTS results show rapid OH* formation on defect-rich CeO_2_ nanorods. This behavior is consistent with vacancy-assisted H_2_O dissociation on ceria^73^ and with the nearly barrierless interfacial activation reported for Ni/CeO_2_.^27^ Together, these findings reconcile previous discrepancies by highlighting that OH* dynamics are governed by vacancy density, interfacial sites, and the prevailing pressure–temperature conditions.

To rationalize the experimental findings, density functional theory (DFT) calculations were carried out to evaluate the energetics of the DCMM on the CeO_2_(111) surface (Figure 6). The interaction of water with the pristine CeO_2_(111) surface was studied first. H_2_O adsorbs exothermically with an energy of *E*_r_(**1** → **2**) = – 0.69 eV, forming hydrogen bonds between its H atoms and surface lattice oxygen (O_lattice_) with a characteristic H···O distance of 2.01 Å, consistent with previous reports (−0.49 to – 0.73 eV).^74^ The dissociation of adsorbed H_2_O into OH* and H* is essentially thermoneutral (*E*_r_(**2** → **4**) = 0.06 eV) and requires a low barrier of 0.12 eV (**3**).^74,81^ These OH* and H* groups serve as active participants in subsequent C–H bond activation steps.^3,59^ In addition, the presence of OH* coverage from dissociated H_2_O enhances CH_4_ adsorption with a slightly higher binding energy up to three dissociated H_2_O molecules at the surface, as shown in Figure S18.

**Figure 6:**
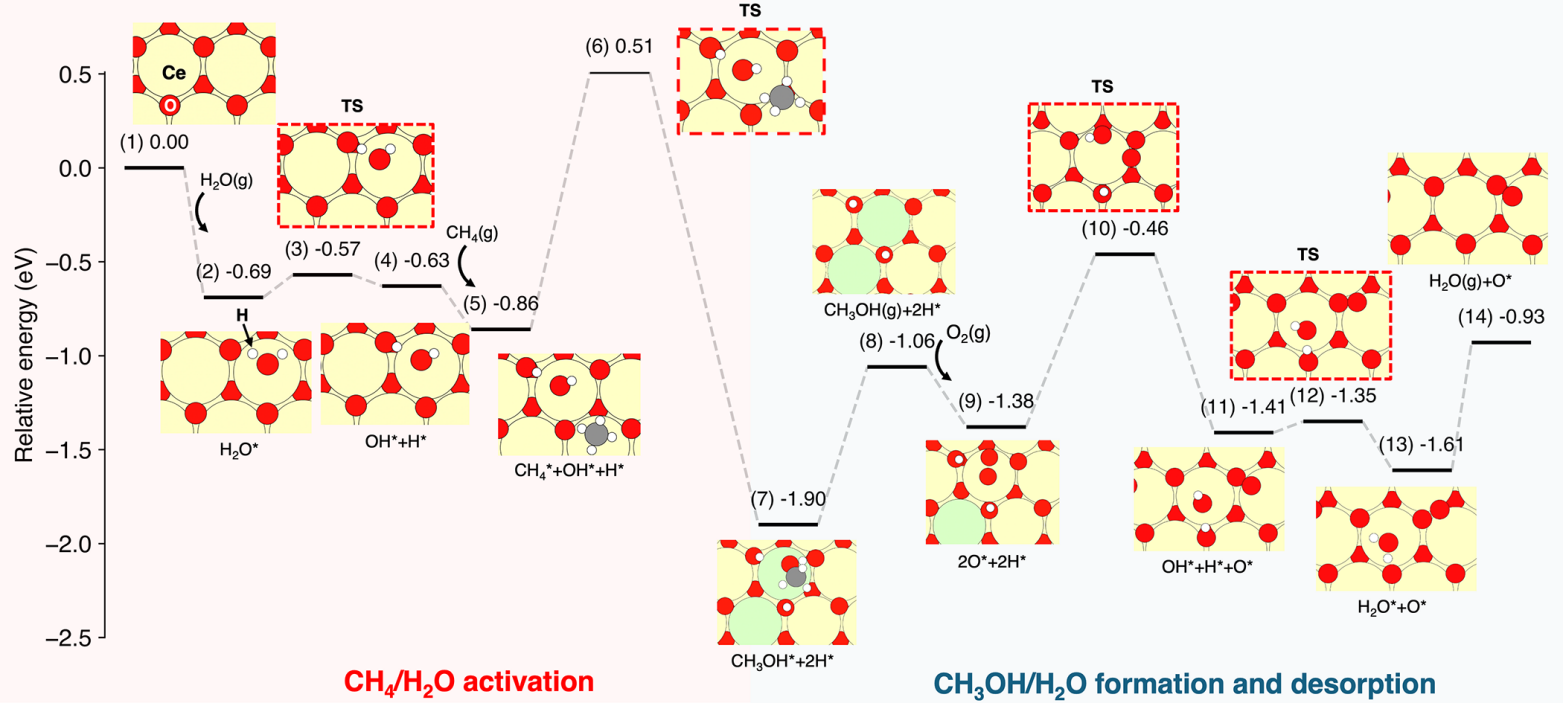
Energy profile of CH_4_ conversion to CH_3_OH on CeO_2_(111). Starting from a bare surface (**1**), an H_2_O molecule adsorbs at a Ce^4+^ site (**2**). The adsorbed H_2_O dissociates crossing a transition state TS (**3**) to form OH* and H* species on the surface (**4**), the latter is generated using a surface lattice oxygen. Then, a CH_4_ molecule adsorbs at the surface (**5**) and dehydrogenates to CH_3_ and H over a barrier (**6**), the former spontaneously forms CH_3_OH with a OH* moiety remaining at the surface (**7**). The desorption of CH_3_OH is shown in step (**8**), leaving 2H* species on the surface. An O_2_ adsorbs at a Ce site (**9**), creating a surface superoxo-like species O_2_^–^*. The O_2_^–^* dissociates to form OH* and O* (**11**) by overcoming TS (**10**). Subsequently, OH* and H* combine to form an H_2_O* species at the Ce^4+^ site (**13**) over barrier (**12**) and subsequently the water molecule desorbs into the gas phase (**14**). Ce^4+^, Ce^3+^, oxygen, carbon, and hydrogen atoms are colored yellow, green, red, gray, and white, respectively.

Subsequently, the interaction of CH_4_ with a hydroxyl species on the surface was modeled. CH_4_ adsorbed atop the Ce site (see Figure 6) with an adsorption energy *E*_r_(**4** → **5**) = −0.23 eV, i.e., slightly more favorable than previously reported for CeO_2_(111), −0.07 eV, likely due to the van der Waals (vdW) dispersion effects included in the present work.^75^ The adsorption energy was also computed on metal/metal–oxide surfaces, CeO_2_/Cu_2_O/Cu­(111) and Ni_4_/CeO_2_(111), resulting in −0.11 and −0.13 eV, respectively.^3,59^

CH_4_ activation to form CH_3_OH with OH* was calculated to be energetically favorable, *E*_r_(**5** → **7**) = −1.04 eV with a barrier height of 1.37 eV (**6**). At step (**5 → 7**), the CH_4_→CH_3_OH conversion consists of two tightly connected steps, C–H activation and CH_3_OH formation. First, the C–H activation of CH_4_* proceeds over a transition state (*E*_a_ = 1.37 eV). After the C–H activation, a metastable CH_3_ radical forms, creating a flat region on the potential energy surface (PES) before the direct combination with OH* to form CH_3_OH (Figure S19). The calculated barrier on the pristine CeO_2_ is 0.40 eV higher than on the bimetallic CeO_2_/Cu_2_O/Cu­(111) surface.^3^ Note that the C–H bond activation barrier of CH_4_ to CH_3_* and H* was reported on a pristine CeO_2_(111) to be around 1.4 eV.^76–78^ Desorption of CH_3_OH into the gas phase was calculated to be endothermic, with *E*_r_(**7** → **8**) = 0.84 eV. After a desorption of CH_3_OH, two H* species remain adsorbed on the surface. O_2_ preferentially adsorbs on the Ce^3+^ site with *E*_r_ (**8 → 9**) = −0.32 eV. Consequently, the Ce^3+^ is oxidized to become Ce^4+^ and the adsorbed oxygen species elongates to 1.33 Å (by 8.13% compared to triplet O_2_ in the gas phase), consistent with a superoxo species O_2_^–^ as reported by Lustemberg et al.^79^ The O* species reacts with O* to form OH*, step (**9 → 11**), which is exothermic with −0.03 eV and a barrier of 0.92 eV. Afterward, H_2_O is generated in thermoneutral fashion by combining H* and OH*, with *E*_r_(**11** → **13**) = −0.20 eV and a small energy barrier of 0.06 eV (**10**). These isoenergetic configurations with a small barrier indicate that both steps may coexist at equilibrium.^74,80^ Finally, H_2_O desorbs into the gas phase with *E*_r_(**13** → **14**)= 0.68 eV, completing the catalytic cycle. Furthermore, the thermoneutrality and low activation barrier of step (**2** → **4**) (the dissociation of water) indicate that H_2_O and OH/H may stably coexist on the surface, providing a reactive environment for subsequent CH_3_OH formation.

Alternatively, we modeled a reaction pathway involving the formation of a lattice oxygen vacancy V_o_, mimicking a Mars–van Krevelen (MvK) mechanism (Figure S20). The formation of H_2_O with lattice oxygen vacancy is strongly endothermic, with an energy of *E*_r_(**8** → **10**) = 1.60 eV and a barrier of 1.59 eV (**9**). A desorption of formed H_2_O on a CeO_2_:V_o_ surface requires *E*_r_(**10** → **11**) = 1.19 eV (0.51 eV higher than on the CeO_2_ surface). However, such a highly endothermic process might still occur experimentally under high temperature conditions. Filling the oxygen vacancy site with O_2_ is highly exothermic with an energy *E*_r_(**11** → **12a**) = −2.66 eV, generating a peroxo-like oxygen species with an O–O bond length of 1.45 Å. Interestingly, an H_2_ molecule can also interact favorably with the Ce^4+^ site, *E*_r_(**11** → **12b**) = −0.57 eV and subsequently dissociate to form O_lattice_–H* and H*, with the latter filling a vacancy site. The calculated reaction energy is *E*_r_(**12b** → **14b**) = −0.16 eV with a barrier of 0.38 eV (**13b**). A charge analysis indicates that H_2_ dissociation follows a heterolytic pathway, yielding a hydride H^–^ and a proton H^+^, which occupy the vacancy site and O_lattice_, respectively.^81,82^ This demonstrates that, in the absence of O_2_, oxygen vacancy sites can be covered by H atoms, hindering further CH_4_ adsorption and activation. As experimentally observed, these calculations highlight the role of a regular supply of O_2_ in replenishing oxygen vacancies and enabling continuous CH_4_ conversion, whereas if only H_2_ is supplied, it may block the surface, preventing C–H bond activation in methane.

1. H_2_O & H_2_ dissociation:
$$H_{2}O^{*}+O_{\mathrm{lattice}}\to {\mathrm{OH}}^{*}+O_{\mathrm{lattice}}H^{*}$$ 1a
$${H_{2}}^{*}+2O_{\mathrm{lattice}}\to 2O_{\mathrm{lattice}}H^{*}$$ 1b
2. Methane initial activation:
$${{\mathrm{CH}}_{4}}^{*}+O^{*}\to {{\mathrm{CH}}_{3}}^{*}+{\mathrm{OH}}^{*}$$ 2a
3. Methyl group further reaction:
  The selective pathway
$${{\mathrm{CH}}_{3}}^{*}+{\mathrm{OH}}^{*}\to {\mathrm{CH}}_{3}{\mathrm{OH}}^{*}$$ 3a
$${{\mathrm{CH}}_{3}}^{*}+{\mathrm{OH}}^{*}\to {\mathrm{CH}}_{3}O^{*}+H^{*}$$ 3b
$${\mathrm{CH}}_{3}O^{*}+H_{2}O\to {\mathrm{CH}}_{3}{\mathrm{OH}}^{*}+{\mathrm{OH}}^{*}+V_{o}$$ 3c
  The over oxidation pathway
$${\mathrm{CH}}_{3}O^{*}\overset{O}{\to }{\mathrm{CH}}_{2}O+{\mathrm{OH}}^{*}\overset{O}{\to }\mathrm{CHO}+2{\mathrm{OH}}^{*}\overset{O}{\to }\mathrm{CO}+3{\mathrm{OH}}^{*}\overset{O}{\to }\mathrm{OCO}+30H^{*}\overset{O}{\to }{{\mathrm{CO}}_{3}}^{2-}+3{\mathrm{OH}}^{*}$$ 3d
4. Replenishment of surface OH:
$$O_{2}+V_{o}\to {O_{2}}^{-}/{O_{2}}^{2-}\to O^{*}+{O_{\mathrm{lattice}}}^{*}$$ 4a
$$H_{2}O+V_{o}\to {\mathrm{OH}}^{*}+H^{*}$$ 4b
$$O_{2}+H_{2}O+V_{o}\to 2{\mathrm{OH}}^{*}+{O_{\mathrm{lattice}}}^{*}$$ 4c

Based on our experimental and theoretical studies, we propose a surface reaction mechanism for the selective oxidation of methane to methanol over CeO_2_, as shown in Scheme 1. The reaction starts with the dissociation of H_2_O and H_2_ on the CeO_2_ surface in the presence of active oxygen (superoxo and/or peroxo), forming surface hydroxyls (OH*) (1a and 1b). However, excessive OH* coverage can block active oxygen sites and inhibit CH_4_ activation, leading to the H_2_ inhibition pathway.

**Scheme 1:**
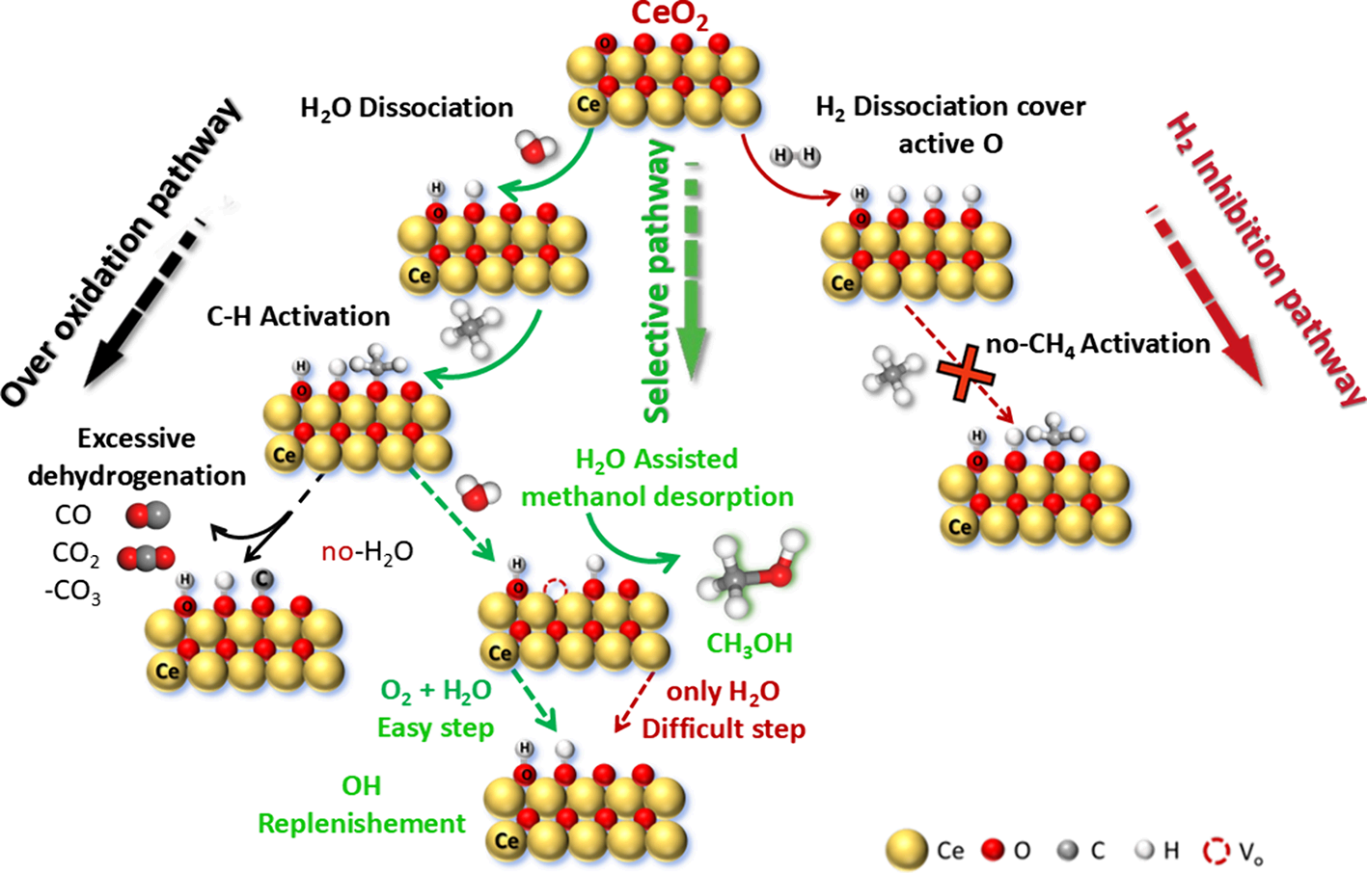
Mechanisms of Direct Conversion of Methane to Methanol (DCMM) on Ceria Surface

Methane activation occurs *via* reaction with surface oxygen, producing CH_3_* and OH* (2). CH_3_* can then react with OH* to form CH_3_OH (3a) or methoxy species (CH_3_O*) (3b). The CH_3_O* species can subsequently react with water to form methanol (CH_3_OH) and regenerate oxygen vacancies (3c), preventing further oxidation and improving methanol selectivity.

In the absence of sufficient water, CH_3_O* may undergo stepwise dehydrogenation to form overoxidized products such as CH_2_O, CHO, CO, CO_2_, and CO_3_^2–^ (3d), following the overoxidation pathway.

To sustain the reaction cycle, oxygen vacancies must be refilled. O_2_ is widely accepted to adsorb/activate as superoxo (O_2_^–^) and/or peroxo (O_2_^2–^) at surface vacancies (V_o_).^83,84^ O_2_ can dissociate at these sites to form active oxygen (4a), while H_2_O alone is less efficient in this role (4b). Importantly, the combined action of O_2_ and H_2_O can more effectively regenerate both OH* and O* species (4c), maintaining catalytic activity.

Overall, water plays a dual role—promoting methanol desorption and assisting in oxygen replenishment—while the control of surface OH* coverage and oxygen availability is key to achieving high methanol selectivity and suppressing side reactions.

Previous UHV model studies, such as CeO_*x*_/Cu_2_O/Cu­(111), reported that ceria alone is inactive for methane activation at lower temperatures.^3^ In contrast, our results demonstrate that pure CeO_2_ powder catalysts exhibit intrinsic activity for DCMM under continuous-flow conditions, underscoring the importance of realistic pressures and defect-rich surfaces in revealing ceria’s catalytic function. Moreover, spectroscopic and theoretical studies have shown that peroxo-mediated O_2_ activation on reduced CeO_2‑x_ surfaces can promote low-temperature CO oxidation but typically favors total oxidation to CO_2_.^79^ Such highly reactive peroxo species may similarly drive overoxidation of methane to CO_2_. However, under wet feed conditions, the relative concentration of peroxo species is suppressed while surface OH is enhanced, thereby shifting the reaction pathway toward partial oxidation of methane to methanol.

## 4 Conclusions

This study demonstrates that pure ceria is capable of catalyzing the direct conversion of methane to methanol (DCMM) under continuous-flow conditions at atmospheric pressure, without the need of noble or transition metals. Steady-state performance tests indicate that CeO_2_ achieves up to 80% methanol selectivity at 300–350 °C, while higher temperatures promote CH_4_ conversion at the cost of selectivity due to overoxidation to CO and CO_2_. Mechanistic insights derived from kinetic analysis, *in situ* DRIFTS, *in situ* AP-XPS, and DFT calculations converge on a redox-mediated pathway in which lattice oxygen and surface OH species cooperatively activate methane and stabilize reactive intermediates. Water plays a dual role: enhancing selectivity by suppressing overoxidation and promoting methanol desorption through OH-mediated proton transfer. Co-feeding O_2_ and H_2_O regenerates both lattice oxygen and OH groups, sustaining the redox cycle and enabling long-term catalyst stability. In contrast, the presence of H_2_ blocks CH_4_ activation by saturating the surface with OH species.

Overall, our findings highlight the potential of metal-free oxide catalysts for selective methane valorization and provide molecular-level guidelines for tuning surface reactivity through the controlled interplay of oxygen and water. Future work should focus on engineering ceria-based materials with tunable surface oxygen reactivity and enhanced redox functionality, including the rational design of mixed oxide systems. In parallel, systematic investigation of reaction pressure effects and the development of reactor configurations optimized for scale-up will be critical for translating these mechanistic insights into practical catalytic technologies.

## Supplementary Material



## Data Availability

All simulation files and experimental data are available on the Materials Cloud platform (DOI: 10.24435/materialscloud:wn-rx).

## Notes

Catalytic performance data under various reaction conditions; structural characterization of CeO_2_ before and after the DCMM reaction (XRD, N_2_ physisorption, and XPS); *in situ* AP-XPS C 1s and O 1s spectra under different reaction conditions; calculated reaction energy profile; catalyst color changes under different reaction atmospheres; colorimetric detection of formaldehyde in liquid products; kinetic curve; product standard curve; carbon balance chart during the reaction process; comparison table of space velocity and product yield; calculation of gas-phase reaction mass transfer limit; comparison of adsorption energy of products on the catalyst surface; relationship between CH_4_ adsorption energy and OH* coverage of dissociated H_2_O; calculated reaction energy profile (PDF)
