# Supplementary material for: Synergy of Oxygen and Water in Ceria-Catalyzed Direct Conversion of Methane to Methanol under Continuous Flow
Source: ACS Catal. 2025 Nov 27;15(24):20496–511. doi: 10.1021/acscatal.5c05829 (PMC12723672; doi:10.1021/acscatal.5c05829)
Supplement: Supplementary file 1 [file cs5c05829_si_001.pdf]

Supplementary Information

Synergy of Oxygen and Water in Ceria-Catalyzed Direct Conversion of Methane to Methanol under Continuous Flow

Wen Li[1,5#], Junjie Shi[1,2*#], Parinya Lewis Tangpakonsab[2#], Bin Zhang[1,5], Thomas Haunold[2], Alexander Genest[2], Nevzat Yigit[2], Leonard Atzl[2], Esko Kokkonen[4], Yong Qin[3*], Günther Rupprechter[2*]

[1]State Key Laboratory of Coal Conversion, Institute of Coal Chemistry, Chinese Academy of Sciences, 030001 Taiyuan, China.

[2]Institute of Materials Chemistry, TU Wien, A-1060 Vienna, Austria.

[3]College of Materials Science and Engineering, Qingdao University of Science and Technology, Qingdao 266042, Shandong, China.

[4]MAX IV Laboratory, Lund University, SE-221 00 Lund, Sweden.

[5]Center of Materials Science and Optoelectronics Engineering, University of Chinese Academy of Sciences, 100049 Beijing, China.

Corresponding Authors: Junjie Shi [junjieshiding@gmail.com], Yong Qin [qinyong@qust.edu.cn], Günther Rupprechter [guenther.rupprechter@tuwien.ac.at]

[#]These authors contributed equally to this work.

Table of Contents

Figure S1. .... S3
Figure S2. .... S4
Figure S3. .... S5
Figure S4. .... S6
Figure S5. .... S7
Figure S6. .... S8
Figure S7. .... S9
Figure S8. .... S10
Figure S9. .... S11
Table S1. .... S12
Table S2. .... S13
Figure S10. .... S14
Figure S11. .... S15
Figure S12. .... S16
Figure S13. .... S17
Figure S14. .... S18
Figure S15. .... S19
Figure S16. .... S20
Figure S17. .... S21
Table S3. .... S22
Figure S18. .... S23
Figure S19. .... S24
Figure S20. .... S25
Note S1 .... S26
Supporting References S27

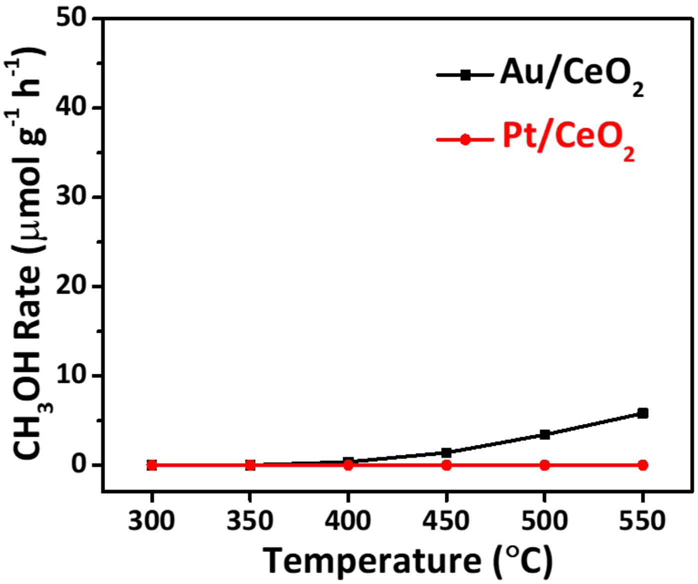

Figure S1. CH_3OH rate as a function of the reaction temperature catalyzed by 2 wt.% Au/CeO_2 and 0.5 wt.% Pt/CeO_2. Reaction conditions: 29 vol.% CH_4 + 53 vol.% H_2O + 9 vol.% O_2 (total flow: 160 mL min^{-1}, catalyst mass: 100 mg, space velocity: 96,000 mL h^{-1} g_{cat}^{-1}).

Au and Pt was loaded on CeO_2 through a modified deposition precipitation (MDP) method. To prepare the 2 wt.% Au/CeO_2 catalyst, 500 mg of CeO_2 powder was added to 1 mL of $HAuCl_4$ (0.01 g mL^{-1}) and KOH mixed solution with pH around 9. After aging at room temperature for 3 h, adding 15 mL ammonia and aging for another 12 h, the suspension was filtered and washed with deionized water several times, until no Cl^- was detected anymore (with $AgNO_3$ solution), then dried at 80 °C overnight. The resulting solid was calcined at 400 °C for 4 h (ramping rate of 4 °C min^{-1}) in the air. To prepare the 0.5 wt.% Pt/CeO_2 catalyst, 500 mg of CeO_2 powder were impregnated in 1 mL of $(NH_3)_4{\cdot}Pt\,(NO_3)_2$ (0.0025 g mL^{-1}) solution at room temperature. The above suspension was aged for 8 h at room temperature, dried at 80 °C overnight and then calcined at 400 °C for 4 h (ramping rate of 4 °C min^{-1}) in the air. The catalytic performance of M/CeO_2 (Au/Pt) was tested in accordance with the procedure for the CeO_2 catalyzed DCMM reaction.

Based on our previous characterization and literature reports, the Au and Pt particles are typically 1-3 nm in size.[1] At this loading and dispersion, Au/Pt addition did not enhance methanol formation but rather decreased selectivity compared to bare CeO_2 (Fig. S1). This behavior is likely due to surface modifications induced by the metal nanoparticles, which increase the concentration of active oxygen species and thereby promote over-oxidation of methane to CO_2.

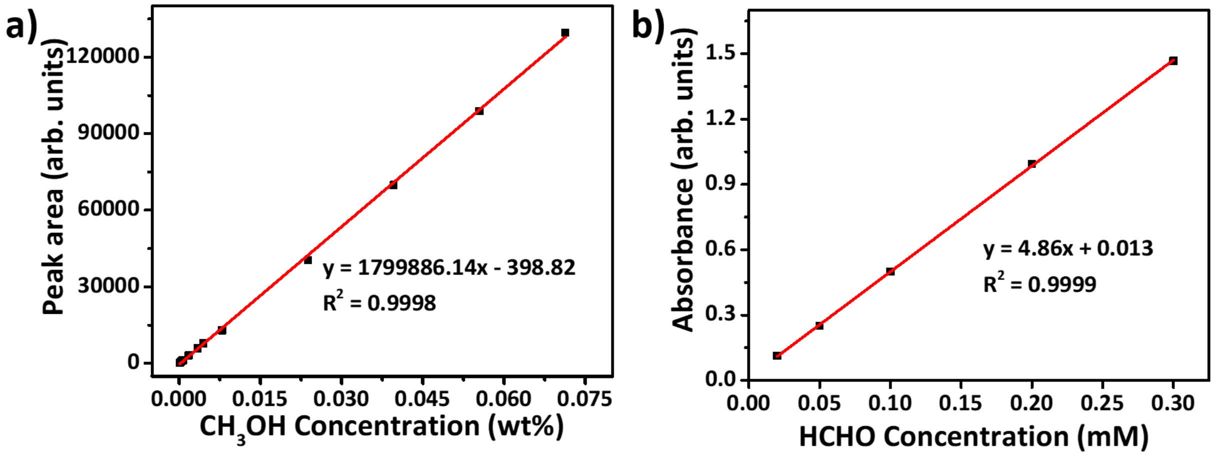

Figure S2. Calibration curves for methanol (a) and formaldehyde (b). Methanol was analyzed by GC, while formaldehyde was detected by UV–Vis spectrophotometry with acetylacetone. Both curves show excellent linearity, and the corresponding fitting equations and R^2 values are included in the graph.

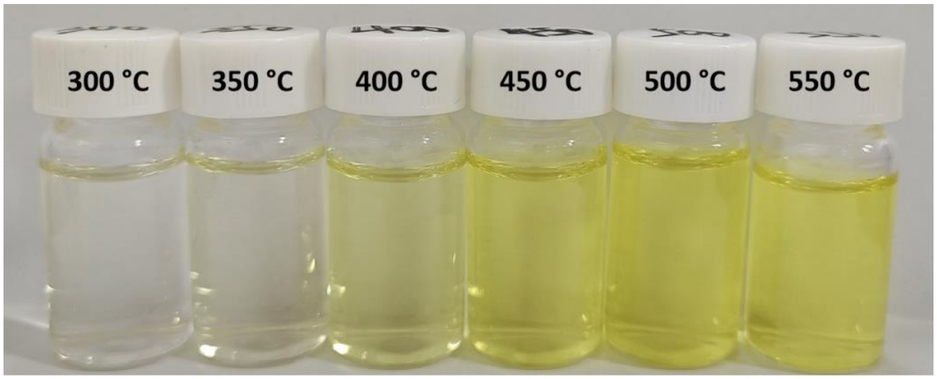

Figure S3. Colorimetric reactions of liquid products with ammonium acetate indicate the presence of formaldehyde. The progressively deepening color from left to right corresponds to increasing formaldehyde concentrations as the reaction temperature rises from 300 to 550 °C. Reaction conditions: 29 vol.% CH_4, 53 vol.% H_2O, and 9 vol.% O_2 (total flow: 160 mL min^{-1}; catalyst mass: 100 mg; space velocity: 96,000 mL h^{-1} g_{cat}^{-1}).

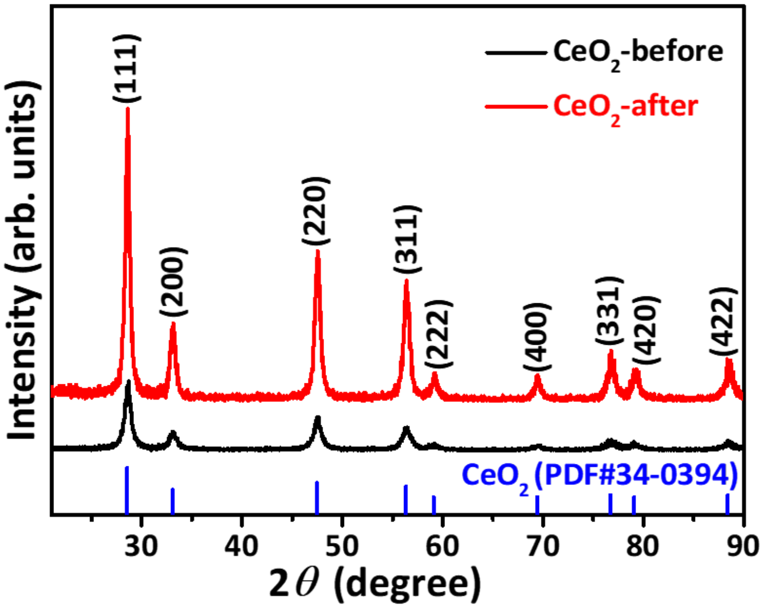

Figure S4. XRD pattern profiles of CeO_2 before and after the DCMM reaction.

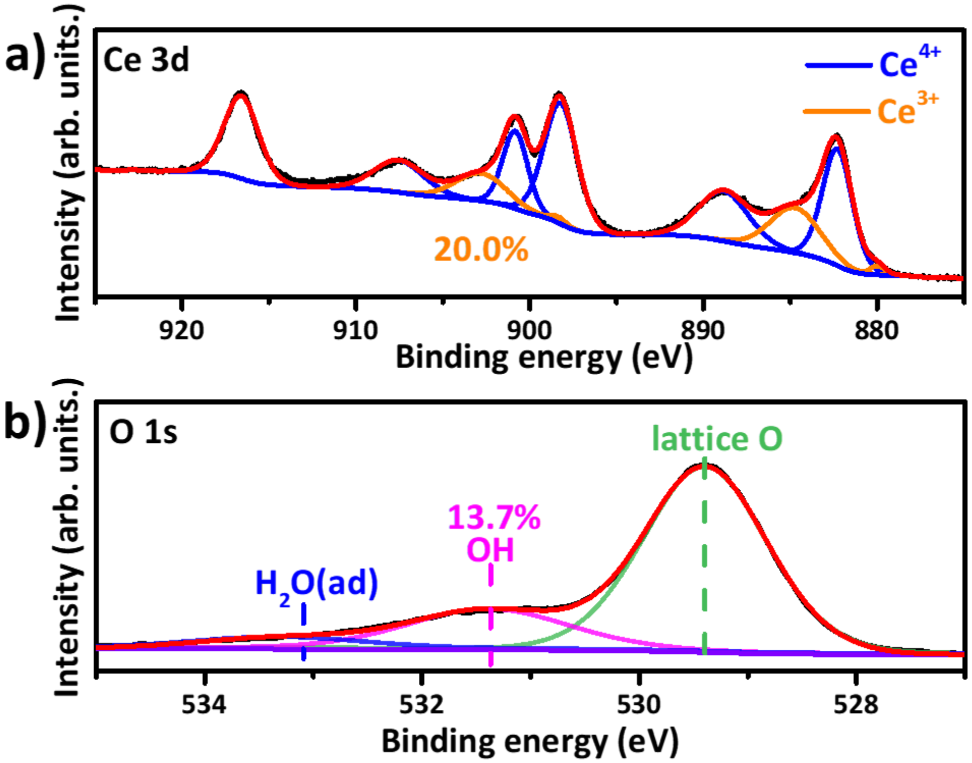

Figure S5. Ce 3d (a) and O 1s (b) XPS spectra of CeO_2 before the DCMM reaction.

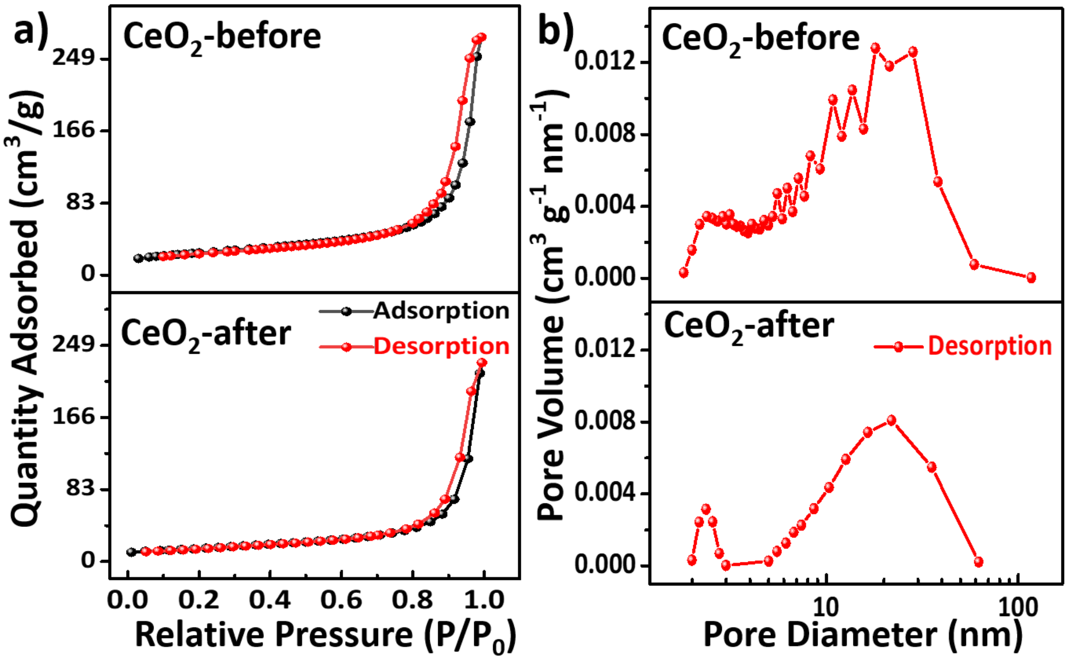

Figure S6. The N_2 adsorption-desorption isotherms (a) and the pore size distribution (b) of CeO_2 before and after the DCMM reaction. Reaction conditions: 29 vol.% CH_4 + 53 vol.% H_2O + 9 vol.% O_2 (total flow: 160 mL min^{-1}, catalyst mass: 100 mg, space velocity: 96,000 mL h^{-1} g_{cat}^{-1}).

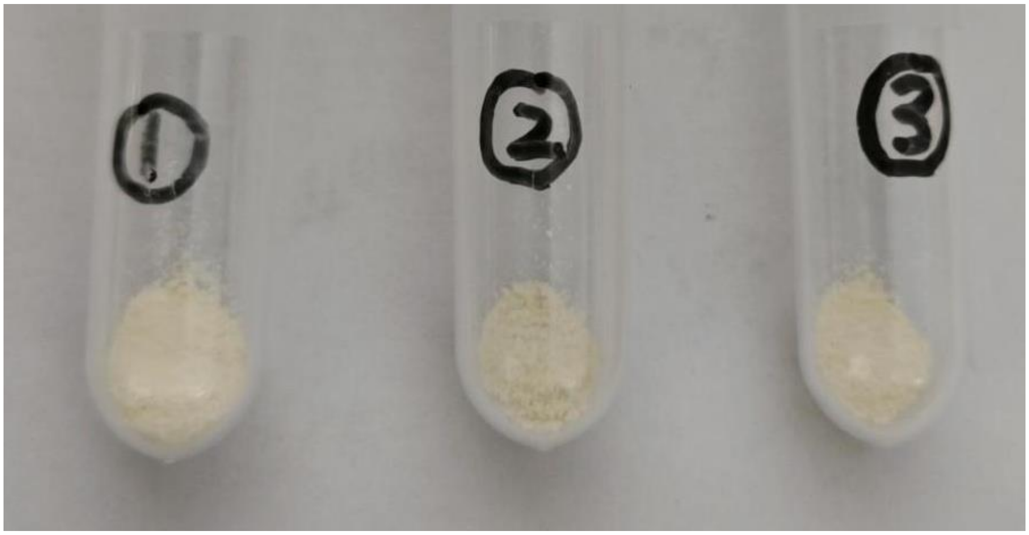

Figure S7. Comparison of the color of the catalysts before the reaction 1) and after 10 h reaction under streams of 2) 29 vol.% CH_4 + 53 vol.% H_2O + N_2, 3) 29 vol.% CH_4 + 53 vol.% H_2O + 9 vol.% O_2 (total flow: 160 mL min^{-1}, catalyst mass: 100 mg, space velocity: 96,000 mL h^{-1} g_{cat}^{-1}).

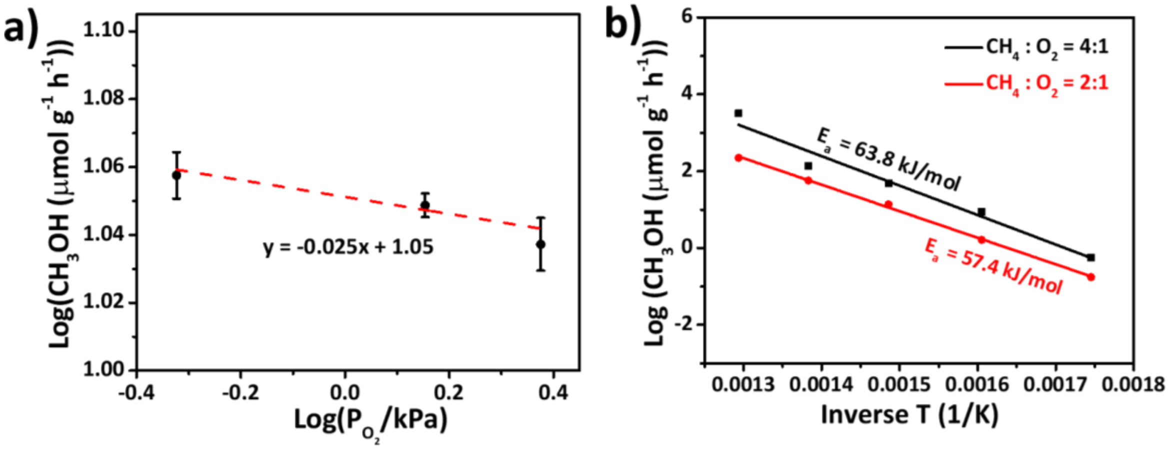

Figure S8. (a) Double-logarithmic plots for determination of reaction orders with respect to O_2 in the low-pressure range (5–24 mbar). Conditions: Temperature = 500 °C, catalyst mass = 200 mg, space velocity = 48,000 mL h^{-1} g_{cat}^{-1}. CH_4 flow fixed at 45 mL min^{-1} while O_2 varied from 0.75 to 3.75 mL min^{-1}. (b) Apparent Activation Energy of CeO_2 calculated from Arrhenius plot under Varying CH_4:O_2 ratios (4:1 and 2:1) in the temperature range of 300-500 °C (total flow: 160 mL min^{-1}, N_2 as balance gas).

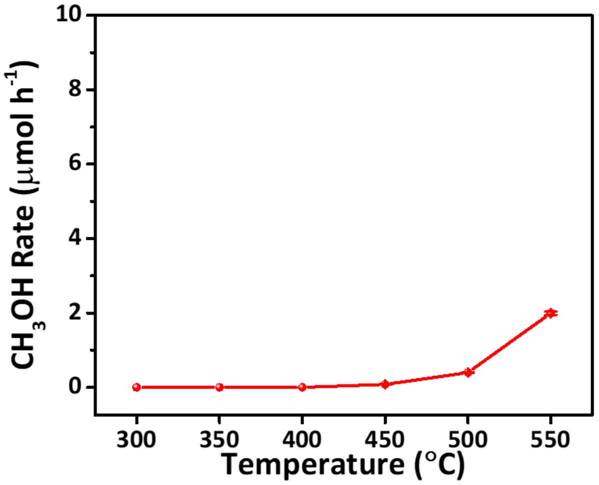

Figure S9. CH_3OH rate as function of reaction temperature without catalyst. Reaction conditions: 29 vol.% CH_4 + 53 vol.% H_2O + 9 vol.% O_2 (total flow: 160 mL min^{-1}, space velocity: 96,000 mL h^{-1} g_{cat}^{-1}).

Table S1. Effect of Catalyst Mass, Contact Time, and space velocity on DCMM Performance under $CH_4 + O_2 + H_2O$ atmosphere at 350 °C.

**Catalyst mass (mg)**	**Bed Height (mm)**	**Contact time (s)**	**Space velocity (mL g^{-1} h^{-1})**	**STY (μmol g^{-1} h^{-1})**	**Normalized STY (μmol m^{-2} h^{-1})**	**CH_4 Conversion (%)**
100	1	0.03	96,000	3.4	0.037	3.9×10^{-4}
200	2	0.06	48,000	2.7	0.030	0.011
500	5	0.15	19200	0.91	0.010	0.034

Reaction conditions: 29 vol.% CH_4, 53 vol.% H_2O, and 9 vol.% O_2 (total flow: 160 mL min^{-1}).

Table S2. Carbon balance data for catalytic reactions under $CH_4 + O_2 + H_2O$ atmosphere in the temperature range of 300–500 °C.

**Temperature (°C)**	**CH_4 in (mol min^{-1})**	**CH_4 out (mol min^{-1})**	**CH_3OH (mol min^{-1})**	**HCHO (mol min^{-1})**	**CO (mol min^{-1})**	**CO_2 (mol min^{-1})**	**C balance (%)**
300	0.001837	0.001804	1.5×10^{-9}	7.7×10^{-10}	0	0	**98**
350	0.001837	0.001834	5.7×10^{-9}	1.7×10^{-9}	0	0	**100**
400	0.001837	0.001824	1.2×10^{-8}	6.3×10^{-9}	0	4.9×10^{-7}	**99**
450	0.001837	0.001775	1.8×10^{-8}	2.1×10^{-8}	0	3.5×10^{-6}	**97**
500	0.001837	0.001793	3.3×10^{-8}	6.1×10^{-8}	2.1×10^{-6}	5.7×10^{-6}	**101**
550	0.001837	0.001545	3.8×10^{-8}	6.3×10^{-8}	5.3×10^{-6}	3.2×10^{-4}	**102**

Reaction conditions: 29 vol.% CH_4, 53 vol.% H_2O, and 9 vol.% O_2 (total flow: 160 mL min^{-1}; catalyst mass: 100 mg; space velocity：96,000 mL h^{-1} g_{cat}^{-1}).

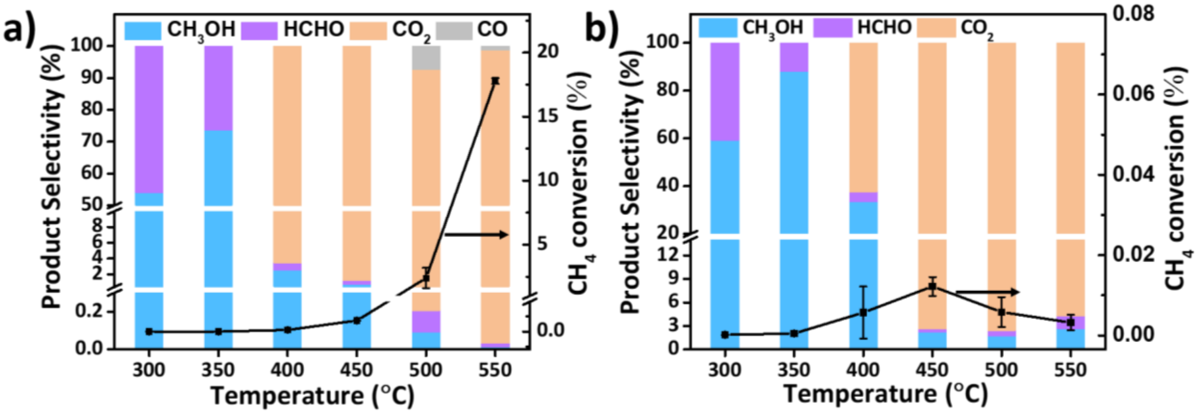

Figure S10. CH_4 conversion and product selectivity as a function of reaction temperature in (a) $CH_4 + H_2O + O_2$ (29:53:9 vol.%) and (b) $CH_4 + H_2O$ (29:53 vol.%). Conditions: total flow = 160 mL min^{-1}, catalyst mass = 100 mg, space velocity = 96,000 mL h^{-1} g_{cat}^{-1}.

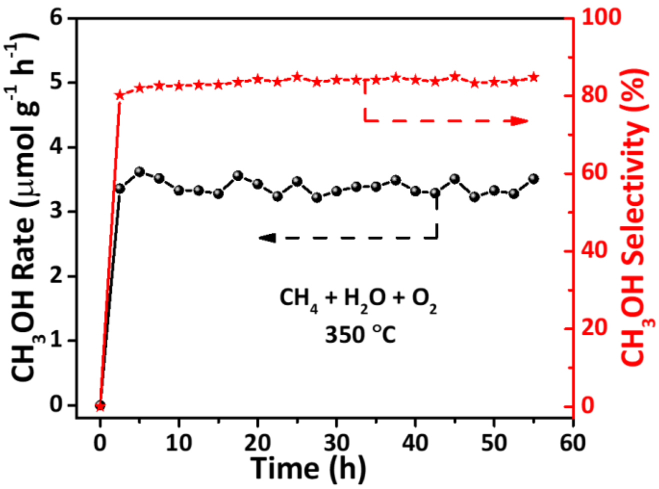

Figure S11. Long-term stability test of CeO_2 in CH_4 + H_2O + O_2 feeds at 350 °C for 55 h on stream. Conditions: total flow = 160 mL min^{-1}, catalyst mass = 100 mg, space velocity = 96,000 mL h^{-1} g_{cat}^{-1}.

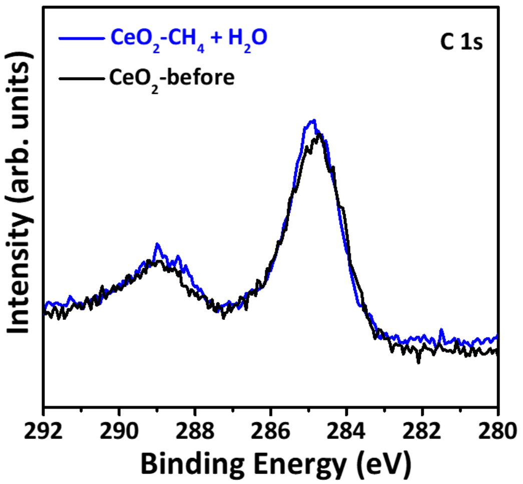

Figure S12. X-ray photoemission (XPS) C 1s spectra of CeO_2 before (black line) and after (blue line) 10 h on-stream at 450 °C under a reaction atmosphere of 29 vol.% CH_4 and 53 vol.% H_2O, balanced with N_2 (total flow: 160 mL min^{-1}, catalyst mass:100 mg, space velocity: 96,000 mL h^{-1} g_{cat}^{-1}).

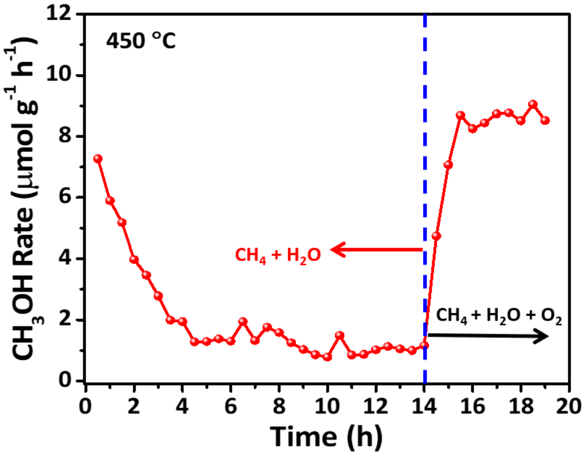

Figure S13. CH_3OH rate as a function of reaction time during a long-term stability test at 450 °C. Feed gas switch from 29 vol.% CH_4 + 53 vol.% H_2O (at 14h) to 29 vol.% CH_4 + 53 vol.% H_2O + 9 vol.% O_2, all feed gases balanced with N_2 (total flow: 160 mL min^{-1}, space velocity: 96,000 mL h^{-1} g_{cat}^{-1}).

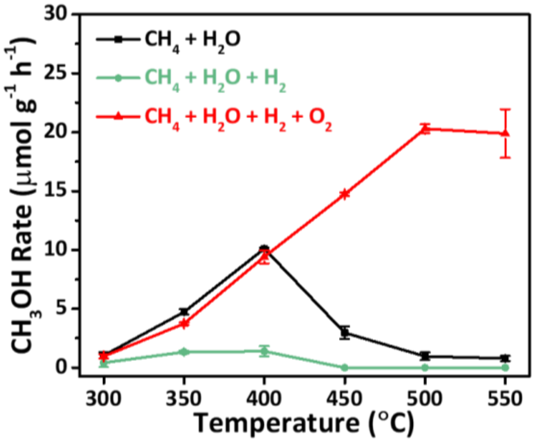

Figure S14. CH_3OH yield as a function of reaction temperature in different reaction atmospheres: 29 vol.% CH_4 + 53 vol.% H_2O (black line), 29 vol.% CH_4 + 53 vol.% H_2O + 9 vol.% H_2 (green line), 29 vol.% CH_4 + 53 vol.% H_2O + 9 vol.% H_2 + 9 vol.% O_2 (red line) at temperatures from 300 to 550 °C, all feed gases balanced with N_2 (total flow: 160 mL min^{-1}, catalyst mass: 100 mg, space velocity: 96,000 mL h^{-1} g_{cat}^{-1}).

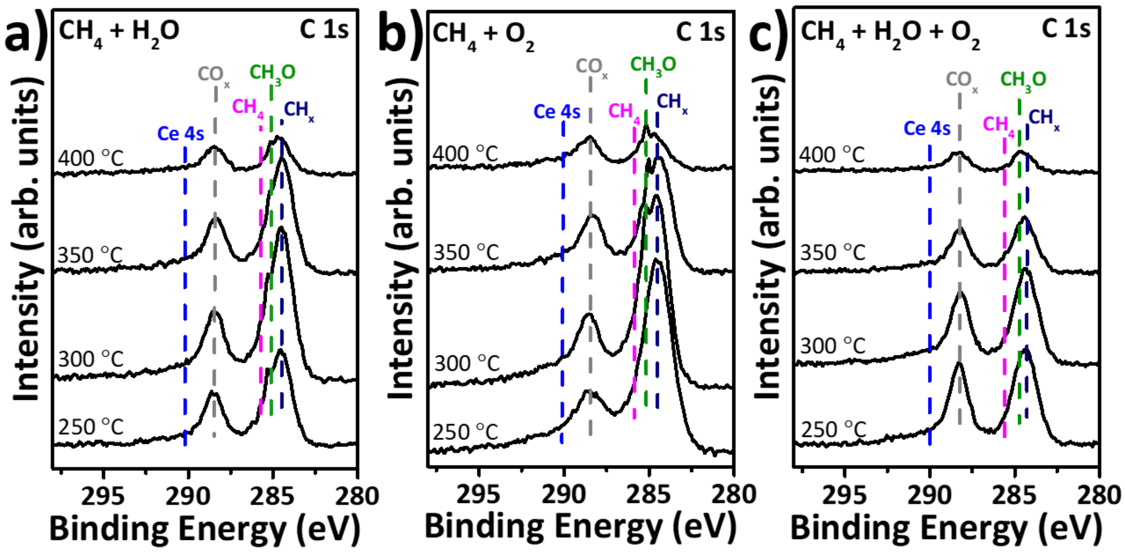

Figure S15. C 1s region of AP-XPS spectra of CeO_2 exposed to different reaction mixtures: CH_4 + O_2 (a), CH_4 + H_2O (b) and CH_4 + H_2O + O_2 (c) at temperatures from 250 to 400 °C.

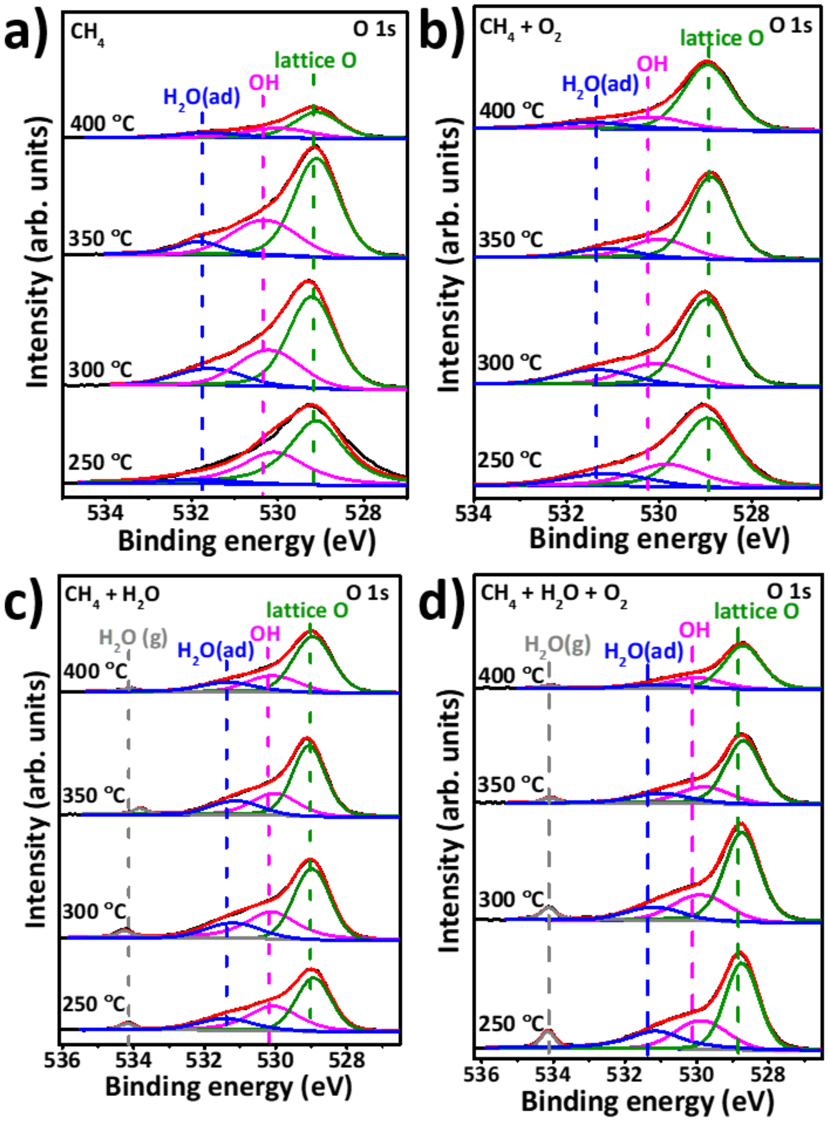

Figure S16. O 1s region of AP-XPS spectra of CeO_2 exposed to different reaction mixtures: CH_4 (a), CH_4 + O_2 (b), CH_4 + H_2O (c) and CH_4 + O_2 + H_2O (d), fitted in the temperature range of 250–400 °C. The O 1s spectra can be resolved into contributions from lattice O (~529.0 eV), OH groups (~530.5 eV), chemisorbed H_2O (~531.5 eV) and gaseous H_2O (~534.0 eV).

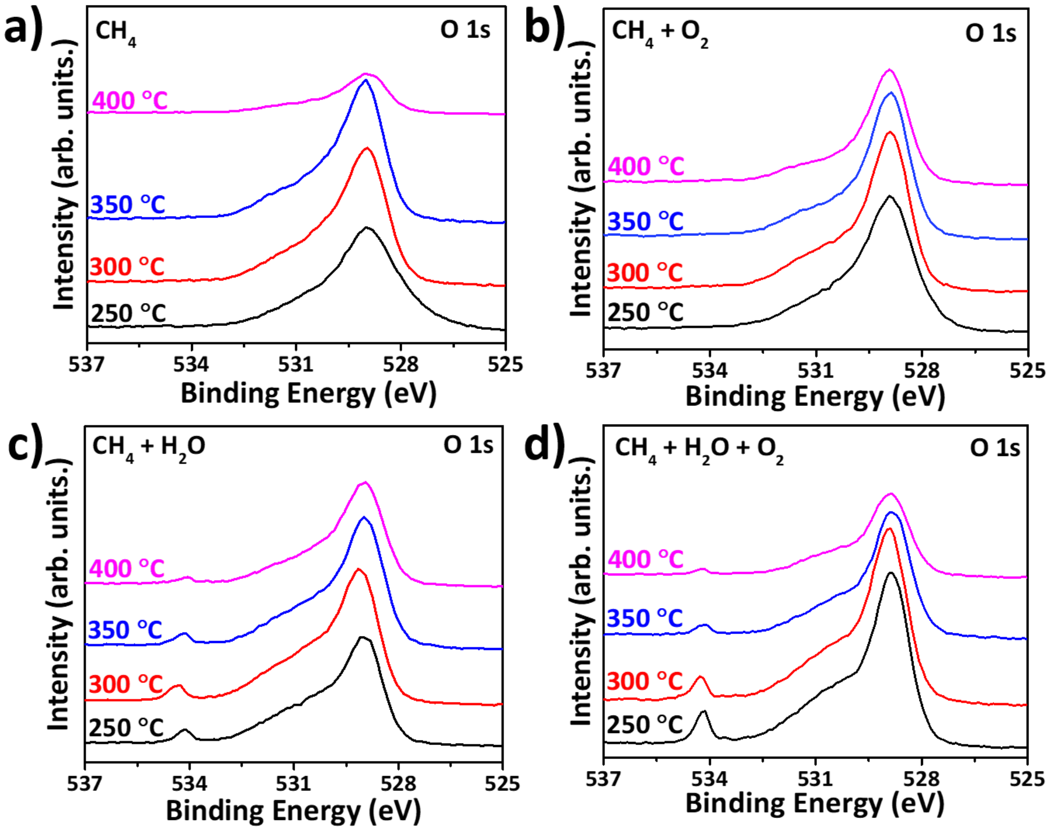

Figure S17. O 1s region of raw AP-XPS spectra of CeO_2 exposed to different reaction mixtures: CH_4 (a), $CH_4 + O_2$ (b), $CH_4 + H_2O$ (c) and $CH_4 + O_2 + H_2O$ (d) at temperatures from 250 to 400 °C.

Table S3. CH_4, CH_3OH, and H_2O (and its dissociation) adsorption energies (in eV) at (111) and (110) terraces of reconstructed CeO_2 and flat CeO_2(111) surface. Calculations using PBE+U–D3 are reported.

Adsorbate	Nanorod		Flat surface
	(111)	(110)	(111)
CH_4	-0.31	-0.24	-0.18
CH_3OH	-0.80	-0.87*	-0.78
H_2O	-0.68	-0.68	-0.69
OH + H	-0.65	-1.04	-0.63

* Adsorption at the edge between the (111) and (110) terraces.

Exploratory DFT calculations on a model nanorod exposing (111) and (110) terraces (Table S3) further show that adsorption of the probe molecules is rather similar on the surfaces at the nanorod and the (111) terrace. Note that a dissociative H_2O adsorption (OH + H) is by 0.4 eV more exothermic on the (110) facet of the nanorod than on the other probed surfaces. This higher exothermicity on the (110) indicates that the consumption of OH* and H* is challenging.

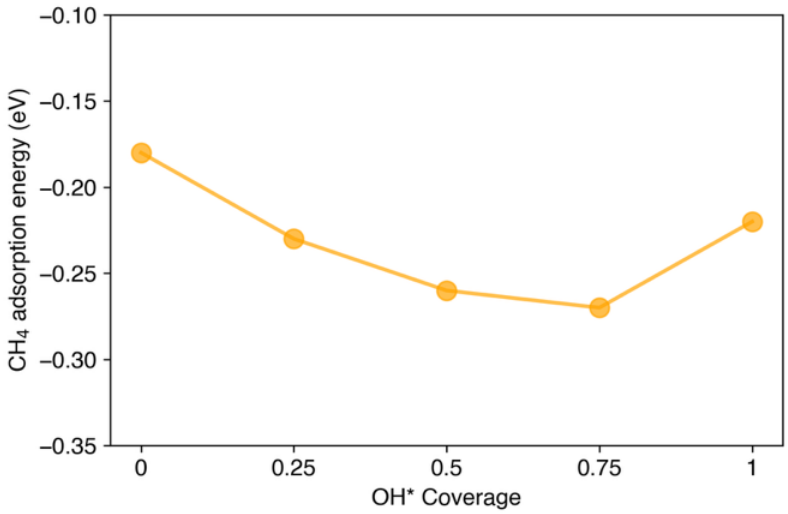

Figure S18. CH_4 adsorption energy as a function of OH* coverage from dissociated H_2O on p(2×2)–CeO_2(111). The adsorption strength increases with hydroxyl coverage up to $\theta = 0.75$. At $\theta = 0.75$ (1), OH* and H* species recombine to form one (two) H_2O molecule(s). The initial position of CH_4 was kept the same for all coverages.

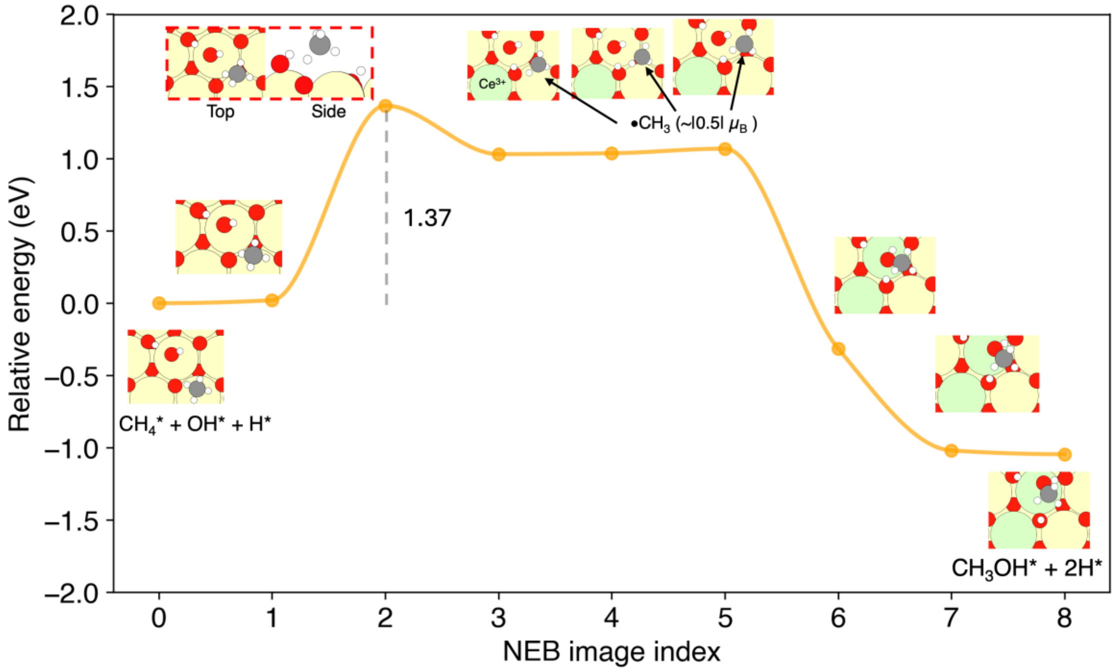

Figure S19. Minimum energy pathway (MEP) of CH_4→CH_3OH. At the transition state ($E_a = 1.37$ eV), a C–H bond of CH_4 is activated. Consequently, a metastable methyl (•CH_3) radical formed with one surface Ce atom being reduced (Ce^{3+}), creating a flat region on the PES (image 3 to 5). Ultimately, CH_3OH forms with two surface Ce^{3+}.

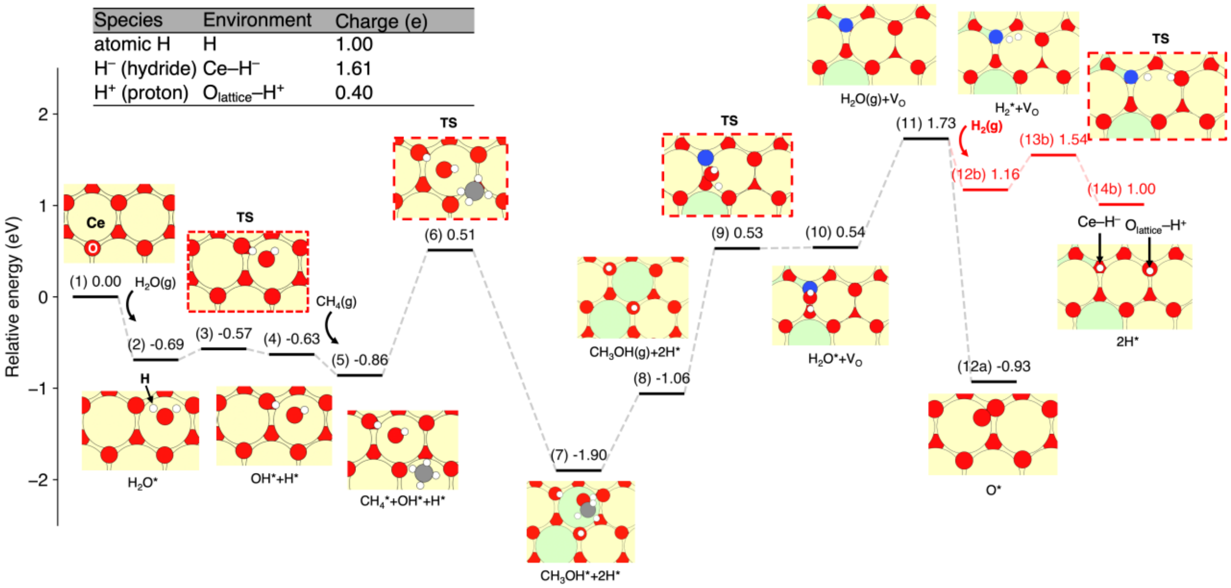

Figure S20. Potential energy surface of CH_4 conversion to CH_3OH at a CeO_2(111) surface following a Mars–van Krevelen mechanism (MvK). The heterolytic dissociation of H_2 forms a hydride H^- species that occupies oxygen vacancy site as well as a proton H^+ that adsorbs at a lattice oxygen $O_{lattice}$, (14b). Ce^{4+}, Ce^{3+}, oxygen, carbon, and hydrogen atoms are color-coded in yellow and green, red, grey, and white, respectively. Blue represents an oxygen vacancy site.

Note S1. Calculation of mass transfer limitations for a gas-phase Reaction in a fixed bed reactor

The Weisz–Prater number was calculated to assess internal mass transfer limitation:

$$C_{WP} = \frac{r' \times R_p^2}{C_s \times D_{eff}} \ll 1$$

where R_p is the nanorod equivalent radius (~5 nm), C_s is the surface concentration of reactant (6.0~4.2 mol m^{-3}), D_{eff} is the effective diffusivity (~1×10^{-6} m^2 s^{-1}) and r' is the observed rate (0.0025~336 mol m^3 s^{-1}).

The Mears criterion was calculated to assess external mass transfer limitation:

$$C_{Mears} = \frac{r' \times R_p}{C_A \times K_c} \ll 0.15$$

where R_p is the nanorod equivalent radius (~5 nm), C_A is the bulk concentration of reactant (6.0~4.2 mol m^{-3}), K_c is the external mass transfer coefficient (200~344 m s^{-1}) and r' is the observed rate (0.0025~336 mol m^3 s^{-1}).

We have estimated both internal and external mass transfer limitations using the Weisz–Prater and Mears criteria[2] over the full temperature range (300–550 °C). The Weisz–Prater modulus was calculated to be between 1.0×10^{-14} and 2×10^{-9}, all much smaller than 1, confirming no internal diffusion limitation. The Mears criterion gave values between 1.0×10^{-14} and 1.2×10^{-9}, also far below the threshold of 0.15, indicating no external diffusion limitation. These results confirm that the observed kinetics are under true kinetic control. For surface-sensitive techniques such as AP-XPS and DRIFTS, all measurements were performed under low-conversion, low-pressure conditions, where transport effects are negligible.

Supporting References

1. Shi, J.; Li, H.; Genest, A.; Zhao, W.; Qi, P.; Wang, T.; Rupprechter, G., High-performance water gas shift induced by asymmetric oxygen vacancies: Gold clusters supported by ceria-praseodymia mixed oxides. *Appl. Catal., B* **2022**, *301*.

2. García-Sánchez, J. T.; Valderrama-Zapata, R.; Acevedo-Córdoba, L. F.; Pérez-Martínez, D.; Rincón-Ortiz, S.; Baldovino-Medrano, V. G., Calculation of Mass Transfer Limitations for a Gas-Phase Reaction in an Isothermal Fixed Bed Reactor: Tutorial and Sensitivity Analysis. *ACS Catal.* **2023**, *13* (10), 6905-6918.
